# Climate-stressed crop mycobiomes as pre-harvest sentinels of human mycotoxin exposure: an AI-enabled One Health framework linking dysbiosis, exposomics, and toxicopathology

**DOI:** 10.3389/fsysb.2026.1921729

**Published:** 2026-08-28

**Authors:** Sayandeep K. Das, Prithviraj Karak, Prachi Parvatikar

**Affiliations:** 1 Department of Pathology, Shri B. M. Patil Medical College Hospital and Research Centre, BLDE (Deemed to be University), Vijayapura, Karnataka, India; 2 Department of Physiology, Bankura Christian College, Bankura, India; 3 Department of Biotechnology, School of Applied Science and Technology, BLDE (Deemed to be University), Vijayapura, Karnataka, India

**Keywords:** aflatoxin, artificial intelligence, climate change, crop mycobiome, human biomonitoring, mycotoxins, one health, toxicopathology

## Abstract

Climate change is altering the conditions under which crop-associated fungi colonize plants and produce mycotoxins. Current control relies on both pre-harvest management and post-harvest testing; however, field-level fungal-community changes are rarely used to guide where and when testing should be intensified. This Review critically compares evidence linking weather and crop stress, fungal-community composition, toxigenic strain activity, contamination during harvest and storage, human biomarkers, and organ-specific effects. On this basis, we propose the Crop Mycobiome-Exposome-Toxicopathology Axis as a testable One Health model, not as a validated diagnostic pathway. Heat, drought, rainfall sequence, insect injury, drying delay, and storage moisture can alter host susceptibility and fungal competition, but their effects differ by crop, fungal guild, and stage of the food chain. Evidence is strongest for climate-linked aflatoxin modelling, maize mycobiome-toxin associations, atoxigenic Aspergillus biocontrol, biomonitoring feasibility, and aflatoxin-related hepatocarcinogenesis. Direct field-to-human datasets remain scarce. We therefore conclude that crop mycobiome measurements may complement, but cannot replace, toxin assays and post-harvest controls. The priority is prospective, paired field-food-human research that tests whether mycobiome indicators improve prediction and intervention timing beyond weather and commodity testing alone.

## Highlights

- Climate and crop stress can alter fungal communities before regulatory mycotoxin limits are exceeded.
- Crop mycobiome dysbiosis is considered a candidate early-warning indicator; it is not a protective “guard” or a substitute for toxin testing.
- Pre-harvest warning must be combined with harvest, drying, storage, segregation, and post-harvest surveillance.
- Human biomonitoring measures internal exposure, but source attribution requires paired food-chain and dietary data.
- Current AI models can support risk triage; the proposed crop-to-human model and Sentinel Mycobiome Risk Index still require prospective validation.

## 1 Introduction: extending mycotoxin control across the pre- and post-harvest continuum

Mycotoxins are a One Health problem because they arise from food- and crop-associated fungi yet affect crop yield, animal feed, milk safety, dietary exposure, occupational exposure, and human disease. WHO emphasizes that moulds can contaminate cereals, dried fruits, nuts, spices, apples, coffee beans, and other foods before harvest, after harvest, during storage, or within the food matrix, and that major mycotoxins can cause acute poisoning, immune effects, cancer, reproductive effects, kidney damage, and developmental risk (World Health Organization, 2023a). This ecological origin and clinical endpoint make mycotoxin risk inseparable from plant health, food systems, veterinary health, environmental change, and public health (World Health Organization, 2023a; European Environment Agency, 2025).

Current surveillance and regulation correctly depend on analytical confirmation of toxins in food or feed. Such testing is indispensable because it determines compliance and supports segregation, rejection, or other lawful action. Its limitation is timing and scope: a result at harvest or in storage identifies the chemical outcome but does not reveal when the crop first became vulnerable or whether storage later amplified field contamination. Earlier ecological indicators could therefore be used to direct sampling and preventive management, but they cannot replace toxin assays or post-harvest controls (European Commission, 2023; Focker et al., 2025; Karlovsky et al., 2016; Stutt et al., 2023).

Climate change makes coordinated pre- and post-harvest control more urgent. Battilani et al. (2016) modelled aflatoxin B1 risk in European maize and wheat across 2254 grid cells and found that mean maize aflatoxin risk increased from 38 under present conditions to 73 under a +2 °C scenario and 95 under a +5 °C scenario, with high-risk sites rising from 20% to 39% and 54%, respectively. The European Environment Agency (2025) also identifies temperature, humidity, and climate variability as factors likely to alter risks from aflatoxins, deoxynivalenol (DON), zearalenone (ZEN), ochratoxin A (OTA), fumonisins, and mixtures in parts of Europe. These sources do not support a uniform increase in every toxin; they indicate regional, crop-specific, and toxin-specific shifts that must be interpreted with crop phenology and storage conditions.

Published studies support three separate observations. Fungal co-occurrence can change aflatoxin, fumonisin, and DON production; pre-harvest maize community profiles have been associated with toxin occurrence under contrasting rainfall; and atoxigenic Aspergillus interventions can reduce aflatoxin contamination (Giorni et al., 2019; Katati et al., 2023; Agbetiameh et al., 2019). These findings justify testing whether community change can provide an earlier warning, but they do not establish a universal sequence in which dysbiosis always precedes toxin accumulation. We therefore use “dysbiosis-before-toxin” as a working hypothesis whose value depends on prospective evidence of temporality and improved prediction.

We use the Crop Mycobiome-Exposome-Toxicopathology Axis to organize climate stress, crop mycobiome instability, toxigenic activation, food-chain handling, human biomonitoring, organ-system toxicopathology, and intervention within one analytical sequence. The purpose is not to present pre-harvest detection as a complete solution. The framework explicitly includes drying, storage, processing, feed transfer, exposure, and biomonitoring because these later stages can amplify, redistribute, or interrupt contamination (Stutt et al., 2023; Karlovsky et al., 2016; Owolabi et al., 2024).

Reading the evidence as a sequence exposes a recurrent weakness in the literature: climate-to-toxin studies, mycobiome surveys, processing studies, and human biomonitoring studies often use different crops, regions, sampling times, and endpoints. Consequently, adjacency between two links should not be mistaken for proof of the full crop-to-human pathway. This Review therefore asks which early states are reproducibly measurable, whether they add information beyond established predictors, and where post-harvest factors override the field signal (Giorni et al., 2019; Katati et al., 2023; Stutt et al., 2023).

Across the available evidence, climate-to-toxin associations and toxin-to-human health evidence are stronger than the direct link between crop mycobiome structure and human biomarkers. We interpret mycobiome dysbiosis as a candidate sentinel only when it can be shown to precede toxin accumulation and improve prediction beyond climate, crop injury, storage measurements, and direct toxin testing. That incremental value has not yet been demonstrated in a single paired field-food-human dataset (Katati et al., 2023; Owolabi et al., 2024).

Four working concepts structure the analysis. “Dysbiosis-before-toxin” describes the proposed early-warning sequence; “microbiome suppression debt” is the authors’ term for loss of protective community functions; the “toxigenic dominance switch” describes a testable change in abundance, activity, or network position; and the Crop Mycobiome-Exposome-Toxicopathology Axis links these field observations to later exposure stages. The first three are interpretive constructs rather than validated measurements, and throughout the Review we distinguish them from findings directly reported in the cited studies.

## 2 Conceptual framework: the Crop Mycobiome-Exposome-Toxicopathology Axis

The Crop Mycobiome-Exposome-Toxicopathology Axis is an organizing model, not a validated causal pathway. It places climate and crop stress, fungal-community change, toxigenic activity, toxin contamination, food-chain handling, human exposure, and organ effects in a common framework while allowing feedback and interruption at each stage. Post-harvest moisture or handling can amplify a modest field signal, whereas rapid drying, sorting, or biocontrol can reduce progression. This is consistent with the One Health view that human, animal, plant, and ecosystem health are interdependent (World Health Organization, 2023b; Stutt et al., 2023).

The framework has seven analytical layers. The climate and crop-stress layer includes heat, drought, rainfall sequence, humidity, salinity, insect injury, and crop development. The crop mycobiome layer includes community composition, diversity within a sample, differences among samples, potentially protective taxa, toxigenic taxa, and interaction patterns. The toxigenic layer separates fungal detection from toxigenic strain identity, toxin-gene potential, gene expression, and measured toxin. The food-chain layer includes harvest, drying, storage, feed transfer, aflatoxin M1 in milk, processing, and contaminated dust. The human exposure layer measures internal dose in urine, blood, serum, plasma, or breast milk. The toxicopathology layer appraises organ-specific evidence. The AI layer is considered only as a method for combining these data, not as an independent source of biological validity (Focker et al., 2025; Owolabi et al., 2024).

For analysis, risk can be separated into six stages: climate and crop stress; mycobiome change; toxigenic potential; measured toxin occurrence; human exposure; and toxicopathology-relevant outcomes. These stages are not assumed to occur in a fixed order in every crop or season. In particular, storage conditions may dominate after harvest, and a field signal may never lead to exposure if effective controls intervene. Staging is useful because it prevents a positive fungal result, a toxin measurement, a biomarker, and a disease endpoint from being treated as equivalent evidence (Focker et al., 2025; Stutt et al., 2023; Owolabi et al., 2024).

A staged model also prevents overinterpretation of any single measurement. Aspergillus detection is not equivalent to aflatoxin production; toxin-gene presence is not equivalent to expression; toxin occurrence in a grain sample is not equivalent to internal dose; and internal dose is not equivalent to disease without considering exposure window, host vulnerability, and co-exposures. The axis therefore separates ecological evidence, molecular evidence, chemical evidence, exposure evidence, and toxicopathological evidence while keeping them biologically connected (Giorni et al., 2019; Lanubile et al., 2021; Vidal et al., 2018).

The authors’ main use of the staged model is hypothesis testing. A candidate mycobiome sentinel should improve prediction of later toxin concentration beyond climate and crop variables. A proposed suppression deficit should remain associated with toxigenic activation after accounting for fungal biomass and weather. Network position should add information beyond relative abundance. Failure of these tests would narrow or reject the proposed constructs rather than merely refine an assumed pathway (Katati et al., 2023; Banerjee et al., 2018; Xiong et al., 2021).

Each stage also supports different action. Climate and crop-stress signals can guide field inspection and sampling. Confirmed toxigenic potential or toxin occurrence can guide intensified testing, harvest planning, rapid drying, segregation, and storage control. Evidence of human exposure can justify targeted biomonitoring and risk communication. These actions are complementary; no early-warning signal removes the need for chemical confirmation or post-harvest management (Agbetiameh et al., 2019; Stutt et al., 2023; Owolabi et al., 2024).

**FIGURE 1:**
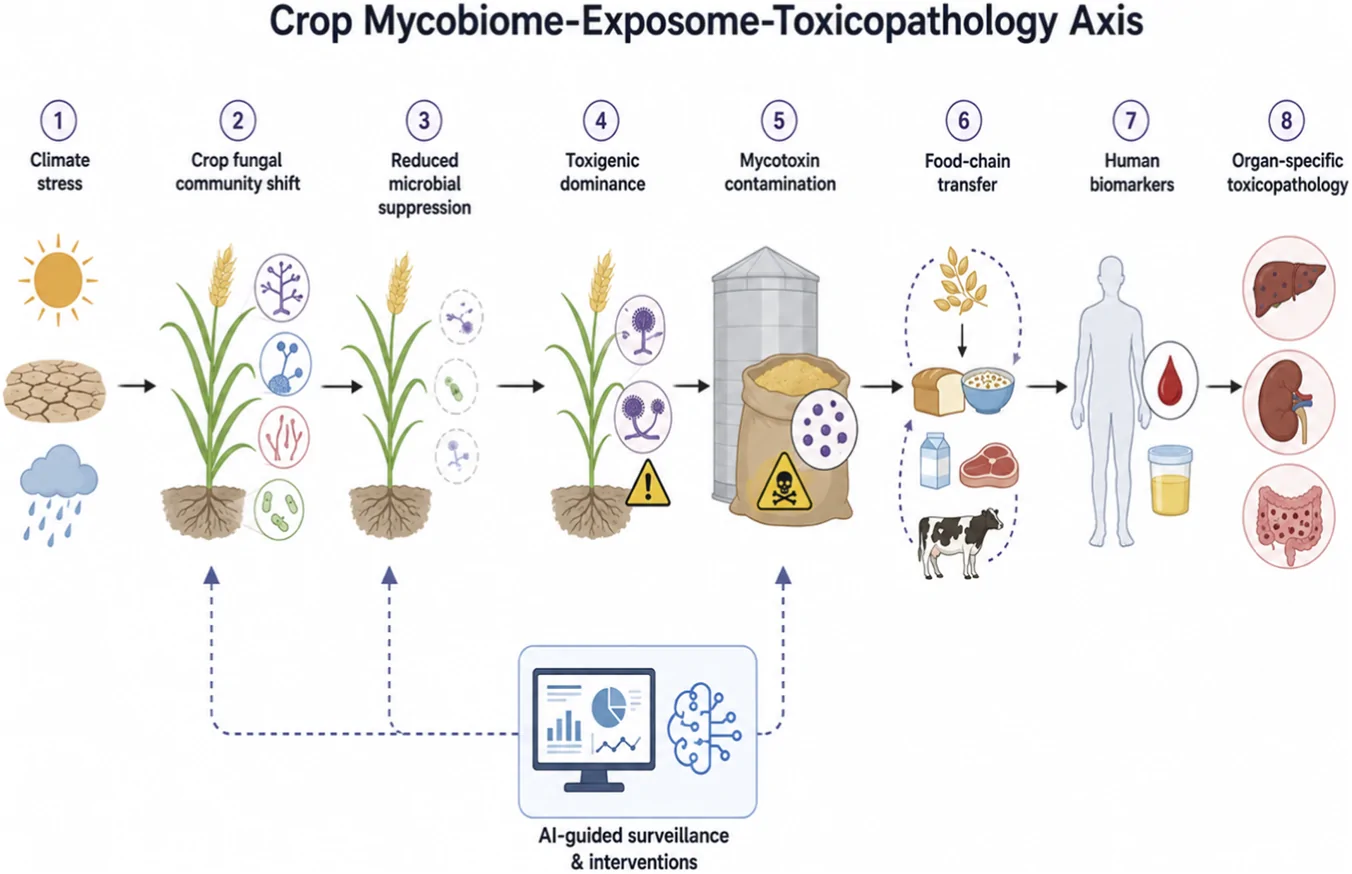
Crop Mycobiome-Exposome-Toxicopathology Axis. The diagram presents an evidence-informed candidate pathway from climate and crop stress to fungal-community change, toxigenic activity, contamination, food-chain transfer, human biomarkers, and organ-specific outcomes. Pre-harvest surveillance is shown as complementary to harvest, drying, storage, and confirmatory toxin testing. The full sequence has not yet been validated in one paired dataset (World Health Organization, 2023a; Katati et al., 2023; Agbetiameh et al., 2019; Stutt et al., 2023; Owolabi et al., 2024).

To reduce jargon and avoid repetition, technical terms are defined at first use in the relevant sections. Figure 1 summarizes the proposed pathway; its arrows indicate an evidence-informed sequence, with several crop-to-human links remaining hypotheses that require paired prospective data.

## 3 Methodological approach for the review

This Review was conducted as a structured framework synthesis with evidence mapping and critical comparison rather than as a systematic review or meta-analysis. The method was selected because the evidence spans crop ecology, climate science, food safety, toxicology, biomonitoring, and computation and cannot be combined in one effect estimate. For each link, we compared the direction and consistency of findings, whether evidence came from laboratory, field, intervention, modelling, or human studies, and whether conclusions were directly observed or inferred across separate literatures (Noyes et al., 2022; White et al., 2020).

The primary question was: how does climate stress alter crop-associated fungal communities and promote mycotoxin emergence, and how can these upstream signals be linked to food-chain exposure, human biomonitoring, organ-system toxicopathology, and AI-enabled prediction? Information sources included PubMed, Scopus, Web of Science, CAB Abstracts, AGRICOLA, Google Scholar for supplementary citation chaining, and authoritative sources from WHO, FAO, EFSA, EEA, Codex, IARC, and national food-safety agencies. Search domains covered crop mycobiome and climate stress, Aspergillus-aflatoxin risk, Fusarium toxins, human biomonitoring, toxicopathology, microbiome-mediated suppression, biocontrol, machine learning, remote sensing, and One Health mycotoxin surveillance (Page et al., 2021; Rethlefsen et al., 2021; Tricco et al., 2018).

Studies were included when they provided climate or environmental data, crop fungal-community data, toxigenic fungal abundance, toxin-gene data, measured mycotoxins, storage or processing information, intervention evidence, human biomarkers, toxicopathology or epidemiological outcomes, or predictive-model validation. Evidence was charted by crop, region, sampling stage, climate variable, fungal taxon, sequencing method, toxin and analytical method, regulatory category, biomarker, organ endpoint, model performance, validation design, and limitation. During synthesis, numerical results were retained only when the source, unit, category, and analytical context could be stated; broad prevalence estimates were treated as context rather than universal thresholds (Page et al., 2021; Rethlefsen et al., 2021; Tricco et al., 2018; Eskola et al., 2020). Searches were iterative and supplemented by backward and forward citation chaining. The literature search and cited-record metadata were updated through 6 August 2026. Because this was a framework review rather than a systematic review, no protocol registration or PRISMA flow diagram is claimed, and the authors do not claim exhaustive retrieval or formal study-level risk-of-bias grading.

Evidence interpretation used a seven-level hierarchy: mechanistic laboratory evidence, field ecological evidence, multi-site surveillance, human biomonitoring, prospective human cohorts, interventions, and integrated crop-human studies. We also recorded disagreement, indirectness, crop and regional specificity, temporal mismatch, and whether a study measured colonization, gene potential, toxin concentration, internal dose, or disease. The strongest evidence currently concerns climate-linked aflatoxin modelling, measured toxin occurrence, atoxigenic Aspergillus biocontrol, biomonitoring feasibility, and aflatoxin-associated hepatocarcinogenesis. Evidence is weakest for the complete field-mycobiome-food-biomarker-organ sequence.

All study selection, interpretation, cross-study comparison, identification of disagreement and gaps, and final scientific wording were reviewed by the authors. In the text, “published studies show” denotes findings reported by cited sources, whereas “we interpret” and “we propose” identify the authors’ synthesis. The proposed suppression debt, full data cube, and Sentinel Mycobiome Risk Index are research constructs, not validated surveillance tools. Reference records were checked for author names, publication year, title, journal, volume and issue, pages or article number, DOI or stable URL, regulatory currency, and one-to-one matching between in-text citations and bibliography entries.

## 4 Major crop-fungus-toxin systems and their climate-sensitive patterns

Before examining individual stages of the axis, it is necessary to separate the major crop-fungus-toxin systems because their climate drivers, timing, processing behaviour, and human evidence are not interchangeable. A single generalized “mycotoxin response to climate change” would obscure these differences.

The Aspergillus-aflatoxin system provides the clearest heat- and drought-sensitive crop-to-cancer example. Aspergillus flavus and A. parasiticus are important aflatoxin producers, but toxin profiles and toxigenicity vary among species and strains in maize, groundnut, tree nuts, spices, oilseeds, and stored commodities. Aflatoxin B1 is strongly linked to hepatocarcinogenesis because it is metabolically activated to DNA-reactive intermediates; aflatoxin M1 can occur in milk after animals consume contaminated feed (World Health Organization, 2023a; National Toxicology Program, 2021; Fountain et al., 2014; Daou et al., 2021; Kang’ethe and Lang’a, 2009).

The Fusarium-fumonisin guild is concentrated around maize and includes Fusarium verticillioides and F. proliferatum, which produce fumonisin B1, B2, and B3. Fumonisin risk is influenced by maize stress, drought, heat, insect injury, and co-occurrence with aflatoxins (Wild and Gong, 2010; Munkvold, 2003). Mechanistically, fumonisins disrupt sphingolipid metabolism by inhibiting ceramide synthase, providing a plausible link to developmental vulnerability, neural tube defects, and other endpoints (Riley et al., 1993; Marasas et al., 2004).

The Fusarium-DON/ZEN guild is associated with wheat, maize, barley, and oats, particularly under humidity and flowering-stage infection. F. graminearum and F. culmorum produce DON and ZEN, with DON linked to gut and immune effects and ZEN linked to estrogenic reproductive effects, especially in animals (World Health Organization, 2023a; European Environment Agency, 2025). The Penicillium/Aspergillus-OTA guild is important in cereals, coffee, dried fruits, grapes, spices, and storage systems; OTA has kidney relevance and possible carcinogenicity, although human causal evidence remains more limited than for aflatoxin (IARC, 1993).

The broader analytical landscape includes alternariol, tenuazonic acid, enniatins, beauvericin, T-2 and HT-2 toxins, patulin, and modified forms; regulatory coverage, routine monitoring, and evidence depth differ substantially among these compounds (World Health Organization, 2023a; European Commission, 2023; EFSA CONTAM Panel, 2011b; EFSA CONTAM Panel, 2014a; EFSA CONTAM Panel, 2014b; Rychlik et al., 2014; Gruber-Dorninger et al., 2017). We interpret this heterogeneity as a reason not to restrict climate-sensitive surveillance to a small set of legacy analytes, while recognizing that exposure and toxicology evidence remains incomplete for several compounds. AI models trained only on routinely measured toxins will reproduce those measurement gaps rather than resolve them. Table 1 summarizes the crop-fungal system-climate matrix.

**TABLE 1:** Crop-fungus-toxin-climate matrix linking major commodities with climate-sensitive toxigenic systems, human evidence, and key limitations (World Health Organization, 2023a; Giorni et al., 2019; Wild and Gong, 2010; IARC, 1993).

Crop	Main fungal guild	Major toxins	Main risk window	Human evidence/boundary
Maize	A. flavus; F. verticillioides	Aflatoxins; fumonisins	Heat, drought, insects; storage moisture	Aflatoxin-HCC is the strongest human anchor; fumonisin developmental evidence is less direct
Groundnut	A. flavus /A. parasiticus	Aflatoxins	Drought, heat, soil contact; storage	Exposure depends on local intake, sorting, and storage control
Wheat, barley, oats	F. graminearum /F. culmorum	DON, ZEN, T-2/HT-2	Moisture near flowering; crop residues	Gut, immune, and endocrine evidence varies by toxin and endpoint
Coffee, dried fruit, grapes	Aspergillus/Penicillium spp.	OTA	Drying and storage humidity	Renal toxicology is important; human causal evidence is limited
Spices	Aspergillus spp.	Aflatoxins; OTA	Drying and storage failure	Contribution depends on concentration, intake, and processing
Milk	Feed-to-milk metabolism	AFM1	Contaminated animal feed	Infant risk depends on measured milk concentration and intake

Source: The systems are simplified for comparison. Species, chemotypes, crop varieties, climate responses, and the strength of human evidence vary by region; the table should not be read as a deterministic crop-to-disease map (World Health Organization, 2023a; Giorni et al., 2019; Wild and Gong, 2010; IARC, 1993).

Comparison across these systems prevents overgeneralization. Aspergillus-aflatoxin risk is often associated with heat, drought, and maize or groundnut stress; Fusarium-DON/ZEN risk is more often associated with flowering-stage moisture and residue ecology; fumonisins overlap with maize stress and insect injury; and OTA frequently reflects drying and storage conditions. We therefore interpret climate change as a potential reshuffling of toxin profiles by crop, region, and food-chain stage, not as a uniform increase in all mycotoxins (World Health Organization, 2023a; European Environment Agency, 2025; Giorni et al., 2019).

### 4.1 Authors’ synthesis

**FIGURE 2:**
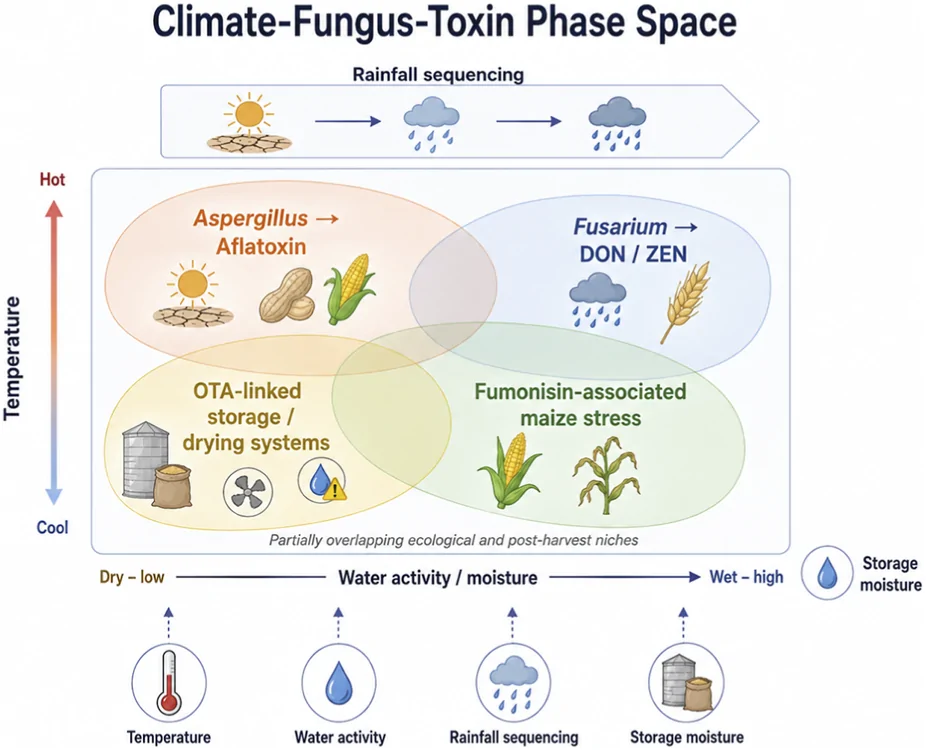
Climate-fungus-toxin phase space. The schematic shows partially overlapping, crop- and stage-dependent niches for Aspergillus-aflatoxin, Fusarium-DON/ZEN, fumonisin-associated maize stress, and OTA-linked drying or storage. Boundaries are conceptual rather than quantitative decision limits (Battilani et al., 2016; Giorni et al., 2019; Katati et al., 2023; Stutt et al., 2023).

The comparative pattern is stronger than any single universal rule: aflatoxin has the clearest hot-dry field signal, DON and ZEN are closely tied to flowering-stage moisture in cereals, and OTA often depends heavily on drying and storage. Consequently, any sentinel index or AI model should be crop-, toxin-, region-, and stage-specific. Figure 2 summarizes these contrasting climate-fungus-toxin niches.

## 5 Climate and crop stress as upstream drivers of mycotoxin emergence

### 5.1 Heat stress and toxin-niche migration

Heat stress can shift fungal biogeography, crop susceptibility, and the relative advantage of thermotolerant fungi such as Aspergillus flavus (Battilani et al., 2016; Fountain et al., 2014; Zingales et al., 2022). Its effect is conditional on crop stage, water availability, insects, competing fungi, and later storage conditions.

Temperature and water activity jointly influence fungal growth and toxin production, but responses vary by isolate, crop, substrate, phenology, and community context. Published thermal ranges are therefore biological guides, not universal field cut-offs or regulatory thresholds (Fountain et al., 2014; Molnár et al., 2023; Zingales et al., 2022).

### 5.2 Drought, humidity cycling, and water activity

Drought can weaken plant resistance, alter kernel physiology, and increase insect-mediated injury, thereby favouring xerotolerant fungi in susceptible systems (Fountain et al., 2014; Dövényi-Nagy et al., 2020). Chauhan et al. (2015) incorporated A. flavus cardinal temperatures of 11.5, 32.5, and 42.5 °C and a crop water supply-to-demand ratio of ≤ 0.2 into a maize aflatoxin model, illustrating how crop water stress can inform pre-harvest risk prediction.

Drought may first increase host vulnerability; later rainfall, delayed drying, or moist storage may restore conditions for fungal growth and secondary metabolism (Molnár et al., 2023; Stutt et al., 2023). The relevant unit is therefore a time-ordered sequence of crop-stage water stress, fungal activity, and storage moisture rather than drought alone.

### 5.3 Altered rainfall and dry-spell ecology

Katati et al. (2023) sampled 40 Zambian maize fields over two seasons and identified 61 fungal genera. Dry-spell conditions were associated with Aspergillus and Penicillium patterns, while Aspergillus and Fusarium relative abundance corresponded with aflatoxin and fumonisin B1 contamination, respectively.

Recent occurrence data reinforce the need to track weather-dependent toxin profiles rather than one toxin in isolation. In 266 maize samples from Serbia and Bosnia and Herzegovina, Janić Hajnal et al. (2026) found regional differences after a hot, dry 2024 season; fumonisins occurred in 88.0% of samples, aflatoxins in 41.4%, and up to ten metabolites co-occurred. Together with the Zambia study, these results support climate-informed, multi-toxin surveillance, but they do not prove that mycobiome change precedes toxin production. That claim requires repeated sampling of the same fields.

### 5.4 Extreme weather, insects, salinity, and crop injury

Extreme weather and insects can damage crop tissues and facilitate fungal entry; insect infestation can also continue after harvest (Pruter et al., 2020; Riungu et al., 2024; Zingales et al., 2022). Salinity remains a plausible modifier of crop stress, but direct field evidence linking it independently to mycotoxin occurrence is limited; it should be tested rather than assumed (Verma et al., 2021).

Across studies, a plausible sequence is drought-related host stress, tissue injury, rewetting, fungal expansion, and post-harvest amplification. The full sequence is not inevitable or yet established as one causal chain. Its practical value is to identify when to sample and where field management, rapid drying, insect control, or storage correction may interrupt risk (Fountain et al., 2014; Riungu et al., 2024; Stutt et al., 2023).

Prediction models should convert raw weather into biologically relevant features such as crop-stage water stress, dry-spell duration, rewetting pulses, canopy wetness, fungal thermal suitability, and storage moisture. These features are closer to host susceptibility and toxin ecology than seasonal averages alone (Chauhan et al., 2015; Battilani et al., 2016; Focker et al., 2025).

The same anomaly can therefore have different effects across crops: heat during maize grain filling may increase aflatoxin susceptibility, whereas moisture around wheat anthesis may favour Fusarium infection. Event sequence, phenology, and fungal niche biology must be modelled together (Giorni et al., 2019; Katati et al., 2023). Table 2 summarizes the study-specific climate-mycobiome-toxin transition anchors discussed above.

**TABLE 2:** Climate-mycobiome-toxin transition anchors relevant to pre-harvest sentinel research (Battilani et al., 2016; Chauhan et al., 2015; Katati et al., 2023; Eskola et al., 2020).

Risk layer	Study-specific anchor	Biological meaning	Source	Authors’ interpretation/limit
European maize aflatoxin risk	Mean index: 38 (present), 73 (+2 °C), 95 (+5 °C)	Warming expands climatic suitability	Battilani et al. (2016)	Useful for spatial prioritization; requires local calibration
High-risk European sites	20% (present), 39% (+2 °C), 54% (+5 °C)	Risk area broadens	Battilani et al. (2016)	Guide sampling, not food-release decisions
A. flavus niche	Warm conditions plus sufficient water activity	Temperature alone is insufficient	Fountain et al. (2014), Molnár et al. (2023)	Growth and toxin responses are context-dependent
Fusarium moisture window	Moisture near cereal flowering	Phenology and wetness are central	Zingales et al. (2022)	No universal temperature cut-off
Mechanistic maize model	11.5/32.5/42.5 °C; water supply:demand ≤ 0.2	Combines fungal biology and crop stress	Chauhan et al. (2015)	Thresholds are model inputs, not regulatory limits
Zambia maize mycobiome	40 fields; 61 genera; rainfall-linked profiles	Community composition carries risk information	Katati et al. (2023)	Association does not prove temporal precedence
Occurrence estimate boundary	Prevalence varies by crop, toxin, region, period, and method	Broad estimates are contextual only	Eskola et al. (2020)	Use local data and analytical limits

Source: Values are study-specific anchors, not universal biological or regulatory thresholds. Comparisons should retain crop, region, sampling stage, analytical method, and uncertainty; broad occurrence estimates should not be applied to an individual commodity without local data (Battilani et al., 2016; Giorni et al., 2019; Chauhan et al., 2015; Katati et al., 2023; Eskola et al., 2020).

#### 5.4.1 Evidence appraisal

Climate-to-aflatoxin modelling is comparatively mature, whereas longitudinal evidence that climate first changes a whole crop mycobiome and that this change then improves toxin prediction remains limited. Direct comparisons of climate-only, crop-injury, mycobiome, and combined models are needed.

## 6 Crop mycobiome dysbiosis: From community change to testable toxigenic risk

A healthy crop mycobiome is not sterile; it contains endophytes, epiphytes, saprophytes, latent pathogens, antagonists, and opportunists. Its relevant property is functional resistance to toxigenic dominance under stress, not richness alone (Porras-Alfaro and Bayman, 2011; Tedersoo et al., 2022).

Here, crop mycobiome dysbiosis means a climate- or management-associated community change accompanied by reduced suppressive function, expansion or activation of toxigenic fungi, or loss of resilience. Candidate measures include evenness, absolute toxigenic fungal biomass, antagonist abundance, toxin-gene load, community turnover, and network structure; each must be validated against measured toxin concentration (Arnault et al., 2023; Banerjee et al., 2018; Xiong et al., 2021).

Alpha diversity describes diversity within a sample, whereas beta diversity describes compositional differences among samples, fields, stages, or conditions (Anderson et al., 2011). Neither metric directly measures suppression or toxin production. Functional claims require toxin outcomes and, where possible, absolute rather than relative fungal measurements.

Network position is one candidate measure of a toxigenic dominance switch, but co-occurrence does not prove interaction. Its value should be tested against simpler predictors such as absolute fungal biomass, toxigenic strain identity, gene expression, and direct toxin measurement (Banerjee et al., 2018; Giorni et al., 2019).

Colonization is not toxigenicity. Detection of Aspergillus or Fusarium does not establish toxigenic strain identity, biosynthetic-gene expression, or toxin accumulation. Species, strain, substrate, crop stage, water activity, injury, fungal competition, and antagonism all modify risk (Giorni et al., 2019; Lanubile et al., 2021; Daou et al., 2021).

Reported suppressive mechanisms include competition, niche occupation, antibiosis, mycoparasitism, induced resistance, and toxin transformation, but evidence is organism- and context-specific (Raaijmakers and Mazzola, 2012; de Lamo and Takken, 2020; Ntushelo et al., 2019). “Microbiome suppression debt” is the authors’ proposed term for a measurable loss of protective function; it is not an established score.

The hypothesis is that similarly stressed fields may diverge because protective taxa or functions persist in one field but not another. Ecological change without toxin quantification indicates potential risk only; longitudinal sampling is required to show whether rewetting, injury, or storage moisture converts that potential into contamination.

**FIGURE 3:**
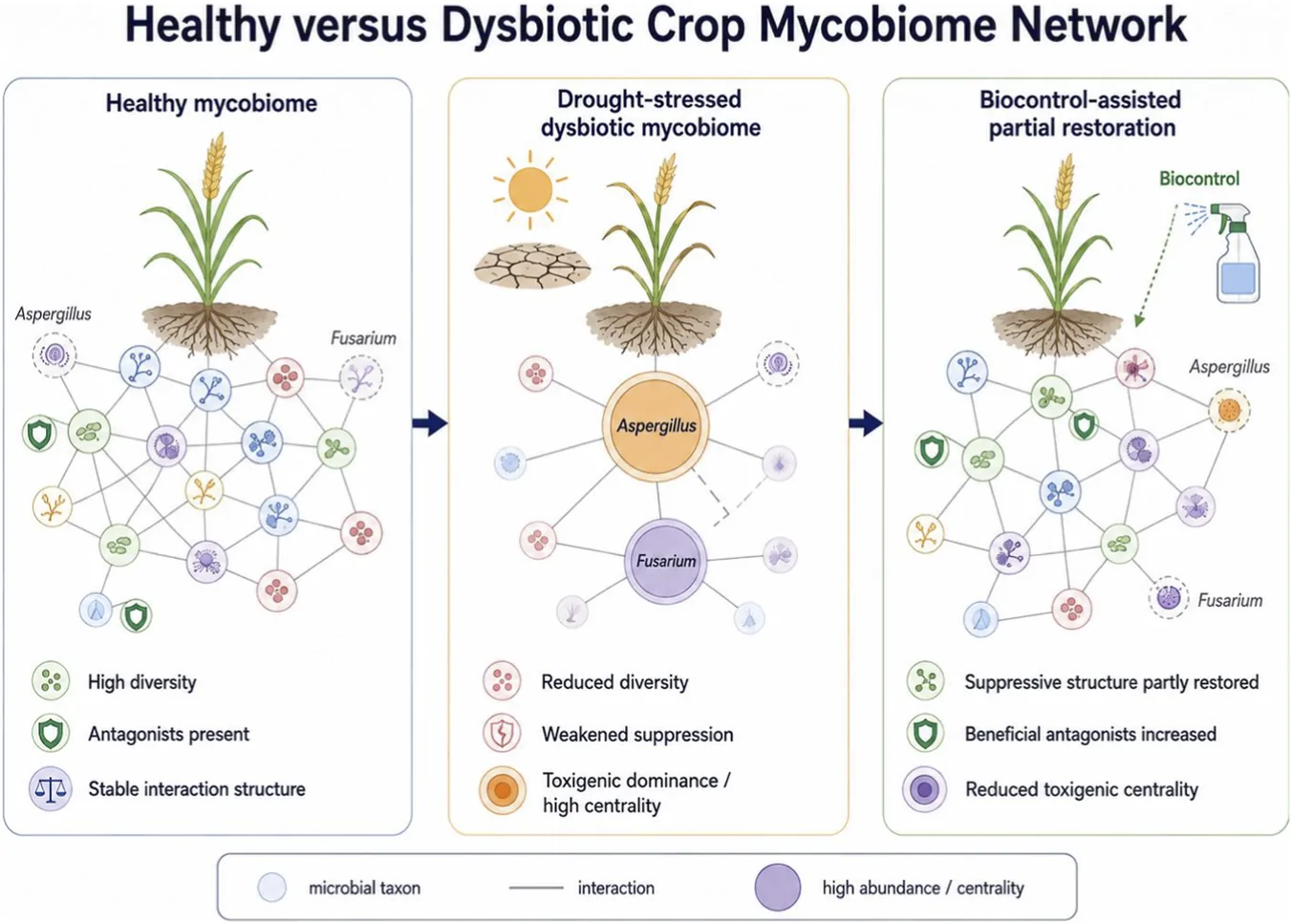
Healthy versus dysbiotic crop mycobiome network. The figure illustrates the authors’ hypothesis that stress may weaken suppressive community structure and increase toxigenic abundance or network position, while biocontrol may partly restore suppression. Network associations do not establish antagonism, toxigenicity, or toxin production (Banerjee et al., 2018; Agbetiameh et al., 2019; Agbetiameh et al., 2020).

Intervention studies offer the strongest test. They should relate changes in antagonist abundance, atoxigenic-to-toxigenic ratios, or network structure to absolute fungal measures and final toxin concentration. These ecological metrics may guide research, but not food-release decisions. Figure 3 illustrates the hypothesized shift from a suppressive to a toxigenic network state and its partial restoration after biocontrol.

Atoxigenic Aspergillus provides the clearest field evidence for community-based control. Selected isolates reduced aflatoxin by 50%–100% in maize and 70%–100% in groundnut trials, while two products tested across 800 Ghanaian farmers’ fields produced 76%–100% lower aflatoxin (Agbetiameh et al., 2019; Agbetiameh et al., 2020).

Other antagonists, including Trichoderma, *Bacillus*, *Pseudomonas*, *Streptomyces*, lactic acid bacteria, yeasts, and endophytes, have shown context-specific activity. Synthetic consortia remain a research option rather than a general recommendation; they should be compared with proven single strains and agronomic or post-harvest controls using persistence, unintended effects, and toxin reduction as endpoints (Raaijmakers and Mazzola, 2012; Ntushelo et al., 2019).

### 6.1 Field case study: biocontrol, mycobiome, and DON in durum wheat

Wachowska et al. (2026) conducted a two-year plot experiment in north-eastern Poland using Debaryomyces hansenii on durum wheat, with Fusarium graminearum inoculation in selected plots. Two high-dose applications reduced Fusarium head blight severity by 79.9% and DON in grain by up to 59.9%. Next-generation sequencing showed changes in the grain and stem mycobiome and an increase in autochthonous yeasts, but the treatment did not eliminate Fusarium colonization. Disease severity, fungal presence, community composition, and toxin concentration therefore did not move in lockstep.

#### 6.1.1 Axis interpretation

The field study directly connects growing conditions, a microbial intervention, mycobiome profiling, and chemical toxin measurement. It supports the ecological-to-chemical segment of the proposed axis while demonstrating why fungal colonization cannot substitute for toxin analysis. Because storage, household food use, human biomarkers, and toxicopathology were not measured, no downstream human effect can be inferred. A stronger extension would follow treated and control grain through storage, consumption, and biomonitoring.

#### 6.1.2 Evidence appraisal

Atoxigenic Aspergillus biocontrol provides strong field evidence for competitive displacement and aflatoxin reduction, while the durum-wheat experiment shows that a microbial intervention can alter disease severity, mycobiome composition, and DON without eliminating Fusarium. These results support ecological intervention, but they do not validate diversity, network centrality, or suppression debt as stand-alone toxin predictors.

## 7 Mycotoxin occurrence and co-occurrence: separating analytical detection, regulatory compliance, and exposure assessment

Analytical detection, regulatory compliance, and exposure assessment answer different questions. A concentration that is quantified above the analytical limit of quantification but below the legal maximum is compliant for that defined food category, yet it can still be included in dietary exposure estimates together with consumption and body-weight data. By contrast, a result below the limit of detection or quantification does not establish a dose and cannot support a claim of chronic biological effect; it should be handled as censored analytical data. Maximum levels are risk-management tools informed by toxicology, exposure, analytical capability, good practice, and food-system feasibility, not simple boundaries between “safe” and “harmful” biology (European Commission, 2023; Wild and Gong, 2010).

EU maximum levels illustrate how food category, intended processing, and population group alter compliance requirements. Table 3 uses selected values from the consolidated Regulation (EU) 2023/915, current to 8 October 2025, to show these distinctions (European Commission, 2023). Lower limits for foods intended for infants and young children reflect additional protection, but simple ratios between adult and infant categories are not biological potency estimates because the products, consumption patterns, and regulatory assumptions are not directly interchangeable. The table therefore emphasizes category conditions and processing context rather than an “infant safety compression factor.” The DON examples incorporate Commission Regulation (EU) 2024/1022, which amended Regulation (EU) 2023/915 from 1 July 2024 (European Commission, 2024).

**TABLE 3:** Selected EU maximum levels with category, processing, and exposure-assessment interpretation (European Commission, 2023).

Toxin	Food category	EU maximum level	Interpretation
Aflatoxin B1	Cereals and cereal products	2.0 μg/kg	Final-food compliance limit; exposure also depends on intake
Total aflatoxins	Cereals and cereal products	4.0 μg/kg	Limit for the aflatoxin sum; not a total-mixture metric
Aflatoxin B1	Maize/rice before sorting or treatment	5.0 μg/kg	Conditional limit for lots destined for sorting or treatment
Total aflatoxins	Maize/rice before sorting or treatment	10.0 μg/kg	Conditional pre-treatment limit; final food must meet its own limit
AFM1	Milk	0.050 μg/kg	Milk-specific compliance limit; intake determines exposure
AFM1	Infant formulae, follow-on formulae and young-child formulae	0.025 μg/kg	Separate infant-formula category; not a biological-effect ratio
DON	Unprocessed cereal grains except unprocessed durum wheat, maize and oats with inedible husk	1000 μg/kg	Applies to the specified unprocessed cereal category
DON	Infant processed cereal-based foods	150 μg/kg	Separate infant cereal-food category; not directly comparable with raw grain
Zearalenone	Unprocessed cereal grains except maize	100 μg/kg	Category-specific limit; endocrine risk is assessed separately
Zearalenone	Unprocessed maize	350 μg/kg	Maize-specific unprocessed category
Fumonisins B1+B2	Unprocessed maize	4000 μg/kg	Unprocessed-maize limit; staple intake belongs in exposure assessment
Fumonisins B1+B2	Maize-based infant foods	200 μg/kg	Separate infant-food category; not a developmental-risk ratio
OTA	Unprocessed cereals	5.0 μg/kg	Unprocessed-cereal limit; not a kidney-effect threshold
OTA	Roasted coffee	3.0 μg/kg	Roasted-coffee category; processing and intake both matter
OTA	Capsicum spices	20 μg/kg	Capsicum-specific category; usual intake is comparatively small

Source: Values are selected examples from the consolidated Commission Regulation (EU) 2023/915 and its amendments. They apply only to the exact food category and processing stage specified in the legal text. Maximum levels are compliance tools, not biological-effect thresholds; exposure assessment additionally requires concentration, intake, body weight, and uncertainty (European Commission, 2023; European Commission, 2024).

Co-occurrence creates an additional exposure-assessment problem. Aflatoxin and fumonisin can occur together in maize, DON and ZEN in wheat or maize, and regulated toxins with emerging metabolites in the same diet (Gruber-Dorninger et al., 2017). However, the presence of several quantified toxins below their individual maximum levels does not automatically prove additive or synergistic harm. Mixture interpretation requires concentration, toxicological mechanism, dose-response information, consumption, and uncertainty; where these data are absent, the appropriate conclusion is a mixture-data gap rather than a presumed effect.

We therefore propose a research-stage mixture exposure profile, not a replacement for regulation. Such a profile could combine quantified concentrations, consumption frequency, commodity dependence, toxicological domains, vulnerable-group context, and available biomarker evidence. Relative potency weights should be used only where supported by risk assessment; otherwise, toxins should be reported by concentration and hazard domain without forced aggregation. The profile would be an analytical aid for paired food and biomonitoring studies, not a legal pass/fail score (European Commission, 2023; Wild and Gong, 2010; Battilani et al., 2020).

For AI, the prediction target must match the intended decision. A compliance model can predict whether a defined maximum level is likely to be exceeded. An exposure model instead requires measured concentrations, consumption, body weight, food-source attribution, and uncertainty. Training only on binary exceedance may overlook variation within the compliant range, but training on an unvalidated mixture score could introduce a different bias. We therefore recommend separate, clearly labelled outputs for compliance risk, quantified exposure, and uncertainty (Focker et al., 2025; Branstad-Spates et al., 2023; Gachoki et al., 2025).

**FIGURE 4:**
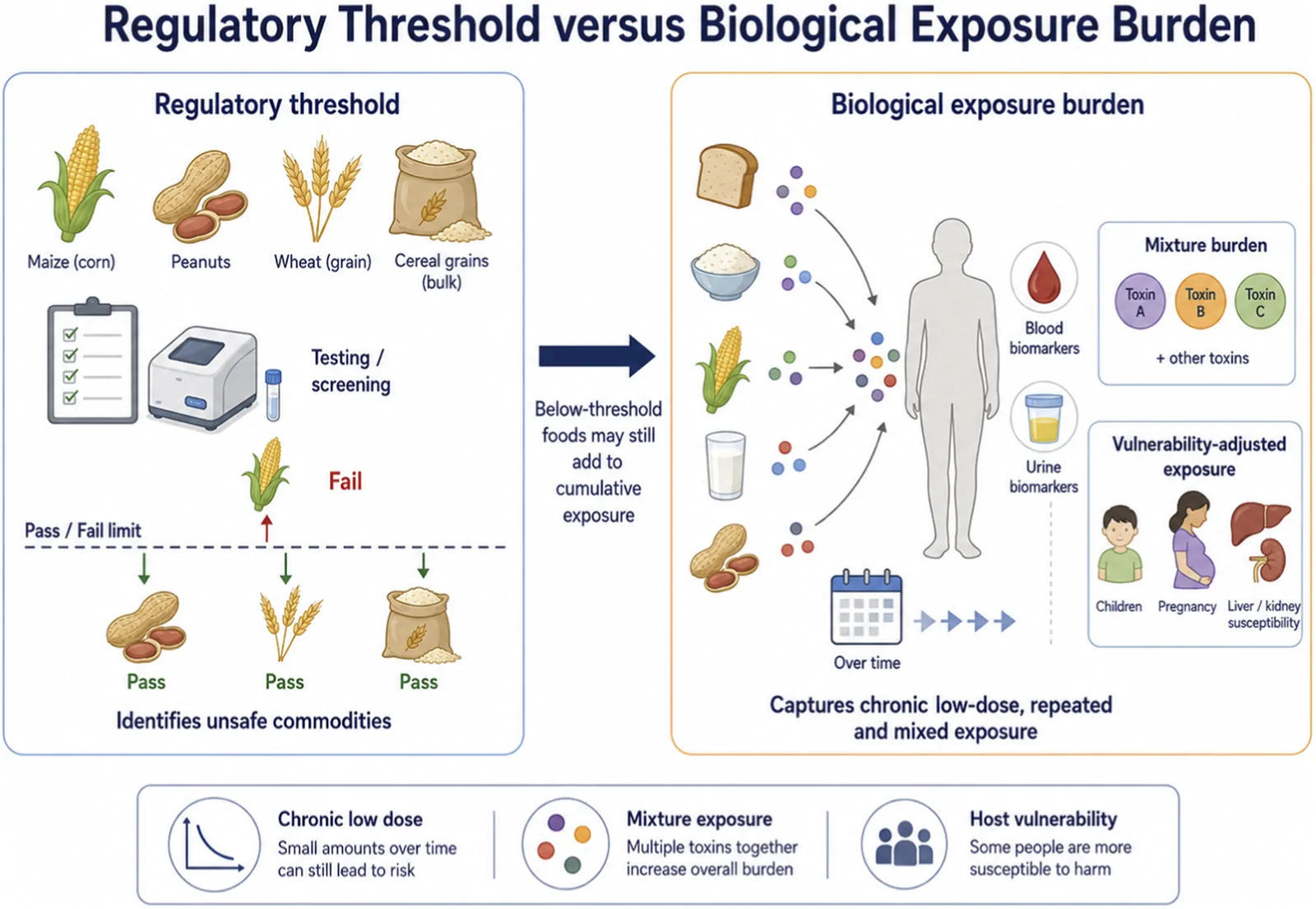
Regulatory threshold versus quantified exposure burden. Regulatory maximum levels identify non-compliant commodities within defined categories. Exposure assessment additionally considers quantified concentrations, repeated consumption, co-occurrence, body weight, and vulnerability. Concentrations below the analytical detection or quantification limit cannot be used to claim a biological dose (European Commission, 2023; Wild and Gong, 2010).

Figure 4 illustrates why a quantified concentration below a legal maximum may still enter cumulative dietary exposure assessment when the food is consumed repeatedly or alongside other contaminated foods. It does not refer to toxins below the analytical detection limit, and it does not imply that compliant food is automatically harmful. The figure complements, rather than challenges, regulatory control by distinguishing legal compliance from exposure estimation (European Commission, 2023; Wild and Gong, 2010).

### 7.1 Authors’ synthesis

Regulation and exposure assessment should be connected but not conflated. Compliance is determined against the applicable legal category; exposure analysis uses quantified concentrations and consumption. A non-detect, a compliant concentration, and a regulatory exceedance must therefore remain separate states in surveillance datasets and AI labels.

## 8 Food-chain amplification and exposure pathways

Pre-harvest mycobiome change can be one candidate early event in a longer contamination pathway, but it is not necessarily the first measurable event and it may be outweighed by post-harvest conditions. Harvest timing, drying delay, mechanical damage, storage moisture, insects, temperature, household storage, milling, processing, feed transfer, and market movement can amplify, redistribute, or reduce contamination (Stutt et al., 2023; Karlovsky et al., 2016). Farm-to-factory modelling of A. flavus and aflatoxin B1 therefore supports a continuum of pre- and post-harvest surveillance rather than a single sampling point (Stutt et al., 2023).

Human exposure routes include direct dietary intake, staple-crop dependence, contaminated infant foods, animal feed transfer to milk and other animal products, breast-milk transfer, occupational exposure among farmers and storage workers, contaminated dust inhalation, and household food insecurity that may force consumption of damaged or visibly mouldy grains (Kang’ethe and Lang’a, 2009; Viegas et al., 2018; Mathew et al., 2025). The most vulnerable groups include infants, children, pregnant people, people with chronic hepatitis B or C, immunocompromised individuals, subsistence farming households, workers exposed to dust, and populations in climate-stressed low- and middle-income regions (Wild and Gong, 2010; European Environment Agency, 2025).

Food processing can reduce mycotoxin concentration through removal of contaminated kernels or fractions, milling redistribution, fermentation, or chemical and enzymatic transformation, but no method guarantees complete removal across toxins and foods (Karlovsky et al., 2016). Industrial maize cleaning has produced substantial aflatoxin reductions, while rejected dust and kernel fractions contained higher concentrations, demonstrating both risk reduction and redistribution rather than disappearance (Pascale et al., 2020). These results support prevention and controlled handling, not a general claim of decontamination.

There is no universally effective decontamination method for all mycotoxins and foods. Early warning should therefore trigger representative confirmatory sampling and preventive actions such as timely harvest, rapid drying, segregation, ventilation, and insect control; it does not mean that every suspected lot must be discarded. Once non-compliance is confirmed, handling must follow the applicable law. In the EU framework, higher limits for lots destined for sorting are conditional, final food must meet the final-consumer limits, and deliberate chemical detoxification of food containing listed contaminants is prohibited (European Commission, 2023).

Food-chain amplification also changes the unit of surveillance. A field sample, a warehouse sample, a market sample, a household meal, and a human urine or blood sample represent different points along the same exposure pathway. The same initial field contamination may produce different public-health consequences depending on drying speed, storage humidity, milling fraction, household sorting, livestock feed diversion, and dietary reliance. A One Health surveillance system should therefore link field-level sentinel signals with storage and consumption data rather than treating them as separate datasets (Stutt et al., 2023; Kang’ethe and Lang’a, 2009; Owolabi et al., 2024).

This linkage is especially important where food insecurity, market pressure, or lack of alternatives makes rejection of an entire lot difficult. The real-world problem is therefore a food-safety and food-security trade-off: suspected lots require representative confirmatory sampling, traceability, and risk-based handling rather than automatic consumption or automatic disposal. Earlier warning has value because it creates time for crop management, rapid drying, segregation, safe storage, and alternative sourcing before households face a contaminated-food decision (Wild and Gong, 2010; Stutt et al., 2023).

## 9 Human biomonitoring and the mycotoxin exposome

Food measurements estimate external contamination; biomonitoring estimates internal dose. This distinction is central to the Crop Mycobiome-Exposome-Toxicopathology Axis because crop contamination becomes clinically meaningful only through absorbed, metabolized, or biologically effective exposure. Urine, blood, serum, plasma, and breast milk are established matrices for different mycotoxins and exposure windows. Hair and nail applications remain exploratory and were not treated as validated core matrices in this Review (Owolabi et al., 2024; Arce-López et al., 2020; IARC, 2015).

Aflatoxin B1-lysine albumin adducts in blood or serum provide a longer-term biomarker of exposure and are particularly relevant to liver cancer studies. Urinary AFM1 reflects recent aflatoxin exposure. Urinary total DON and DON-glucuronides reflect recent dietary exposure, while OTA in serum or plasma may persist longer due to protein binding (Arce-López et al., 2020; Vidal et al., 2018). Multi-mycotoxin LC-MS/MS and high-resolution mass spectrometry panels are increasingly important because they can detect multiple toxins and metabolites within the same biological matrix (Owolabi et al., 2024; Braun et al., 2020).

Population biomonitoring demonstrates exposure outside acute outbreaks. In 360 German 24-h urine samples, total DON was above the limit of quantification in 99%, with a median of 4.3 μg/L (Schmied et al., 2023). In the HBM4EU aligned studies, 1,270 adults from six countries were assessed and 12.3% exceeded the 23 μg/L guidance value, with substantial country variation (Namorado et al., 2024). These studies show feasibility and exposure heterogeneity, but neither identifies the responsible crop, field, or storage stage without paired food-chain data.

Biomonitoring should be interpreted together with toxicokinetics. Urinary biomarkers are useful for recent exposure but may miss longer-term burden when sampling is poorly timed. Blood or serum markers such as AFB1-lysine adducts and OTA can cover longer windows but require stronger laboratory infrastructure. Breast-milk studies are important for infant exposure and require informed consent, privacy protection, careful return of results, and access to risk communication or safer alternatives. These are participant-protection and implementation requirements, not reasons to avoid public-interest biomonitoring. Matrix selection should follow the toxin, exposure window, population, and intervention question rather than analytical convenience alone (Arce-López et al., 2020; Vidal et al., 2018; Cramer et al., 2026).

The exposome layer also creates an objective bridge between crop risk and pathology. A field-level model may predict a high-risk harvest, but human biomonitoring can show whether that risk became internal dose. Conversely, biomarker positivity in a community can trigger backward investigation of storage, diet, feed, and crop mycobiome conditions. This two-way linkage is what converts the framework from an agricultural model into a One Health surveillance system (Owolabi et al., 2024; Schmied et al., 2023; Stutt et al., 2023).

The missing translational dataset is not crop toxin data alone or human biomarker data alone, but paired field-food-human biomonitoring data. Table 4 maps key biomarkers to matrices, exposure windows, and interpretation, making biomonitoring the bridge between pre-harvest warning and toxicopathological relevance (Owolabi et al., 2024; Cramer et al., 2026; Focker et al., 2025).

**TABLE 4:** Human biomonitoring matrix linking toxins, biomarkers, matrices, exposure windows, and interpretation (Arce-López et al., 2020; Vidal et al., 2018; Owolabi et al., 2024; Cramer et al., 2026).

Toxin	Biomarker and matrix	Window	Evidence anchor	Interpretation/limit
Aflatoxin B1	AFB1-lysine albumin adduct; blood/serum/plasma	Longer-term	Widely used in human studies	Established exposure biomarker with strong relevance to aflatoxin-HCC research
Aflatoxin	AFM1; urine or breast milk	Recent	Qidong cohort: Urinary AFM1 >3.6 ng/L; 3.3-fold HCC risk among chronic HBV-positive men	Strong translational anchor; the cohort did not include a paired source-crop dataset
DON	Total DON; urine	Recent dietary	German ESB: 99% above LOQ, median 4.3 μg/L; HBM4EU: 1,270 adults, 12.3% above 23 μg/L	Population surveillance; source attribution requires paired food data
OTA	OTA or OTalpha; serum/plasma, urine, breast milk	Longer than many toxins	Detected in human blood and milk	Exposure marker with renal relevance; developmental interpretation remains uncertain
Fumonisin B1	FB1 or sphinganine:sphingosine ratio; urine/blood	Recent exposure/effect	Mechanistic link to sphingolipid disruption	Candidate effect marker with limited routine human validation
Zearalenone	Alpha-ZEL, beta-ZEL, or ZEN metabolites; urine/serum	Recent	Endocrine-disruption relevance	Supports exposure assessment; human reproductive and developmental inference remains limited
Multi-mycotoxin mixtures	Multi-analyte LC-MS/MS panel; urine, serum/plasma, or breast milk	Variable	Multi-analyte biomonitoring is increasingly used	Needed for exposome-level inference

Source: Biomarker choice must consider toxicokinetics, matrix specificity, sample timing, analytical validation, and vulnerability group (Arce-López et al., 2020; Vidal et al., 2018; Owolabi et al., 2024; Cramer et al., 2026).

### 9.1 Evidence boundary

A biomarker can confirm internal exposure within its toxicokinetic window, but it cannot identify a specific field or commodity without linked dietary, storage, and food measurements. Ethical safeguards, consent, privacy, and return-of-results plans are therefore part of rigorous study design, not obstacles to public-health improvement.

## 10 Human toxicopathology: organ-system mapping of crop-derived fungal metabolites

Aflatoxin B1 provides the strongest human toxicopathology anchor. Its bioactivation produces DNA-reactive intermediates, and hepatitis B infection modifies hepatocellular carcinoma (HCC) risk (IARC, 2002; National Toxicology Program, 2021; Groopman et al., 2021). In a Qidong cohort of chronic hepatitis B-positive men, urinary AFM1 above 3.6 ng/L was associated with a 3.3-fold higher HCC risk (Sun et al., 1999).

Evidence for other toxins is important but less complete in humans. OTA has strong experimental renal toxicology but limited causal human evidence; DON primarily affects gut and immune tissues in mechanistic and animal studies; and ZEN has estrogenic and reproductive effects that are best established in animals (IARC, 1993; Bui-Klimke and Wu, 2015; Pinton and Oswald, 2014; EFSA CONTAM Panel, 2011a).

Fumonisin B1 inhibits ceramide synthase and disrupts sphingolipid metabolism, providing mechanistic support for developmental concern, including neural tube defects (Riley et al., 1993; Marasas et al., 2004). Alternaria toxins, enniatins, beauvericin, and modified forms remain less well characterized for human exposure-response and organ outcomes (EFSA CONTAM Panel, 2011b; EFSA CONTAM Panel, 2014a; EFSA CONTAM Panel, 2014b).

The organ map must therefore be read as an evidence gradient, not as a set of equal causal claims. Aflatoxin-HCC has the strongest human evidence; OTA kidney effects, DON gut and immune effects, ZEN endocrine-reproductive effects, and fumonisin developmental effects vary in directness, biomarker validation, and dose-response resolution.

Mixture occurrence is common, but human mixture toxicology remains less developed. Different toxins may converge on oxidative stress, barrier injury, immune modulation, endocrine signalling, mitochondrial stress, or developmental vulnerability, yet combined effects depend on dose, timing, and host susceptibility (Battilani et al., 2020; Gruber-Dorninger et al., 2017).

The practical gap is a lack of prospective studies linking measured mixtures and validated internal-dose biomarkers to early organ endpoints in vulnerable populations. Until such data exist, the framework assigns each toxin to its best-supported domain and states the uncertainty rather than inferring disease from crop contamination alone.

**FIGURE 5:**
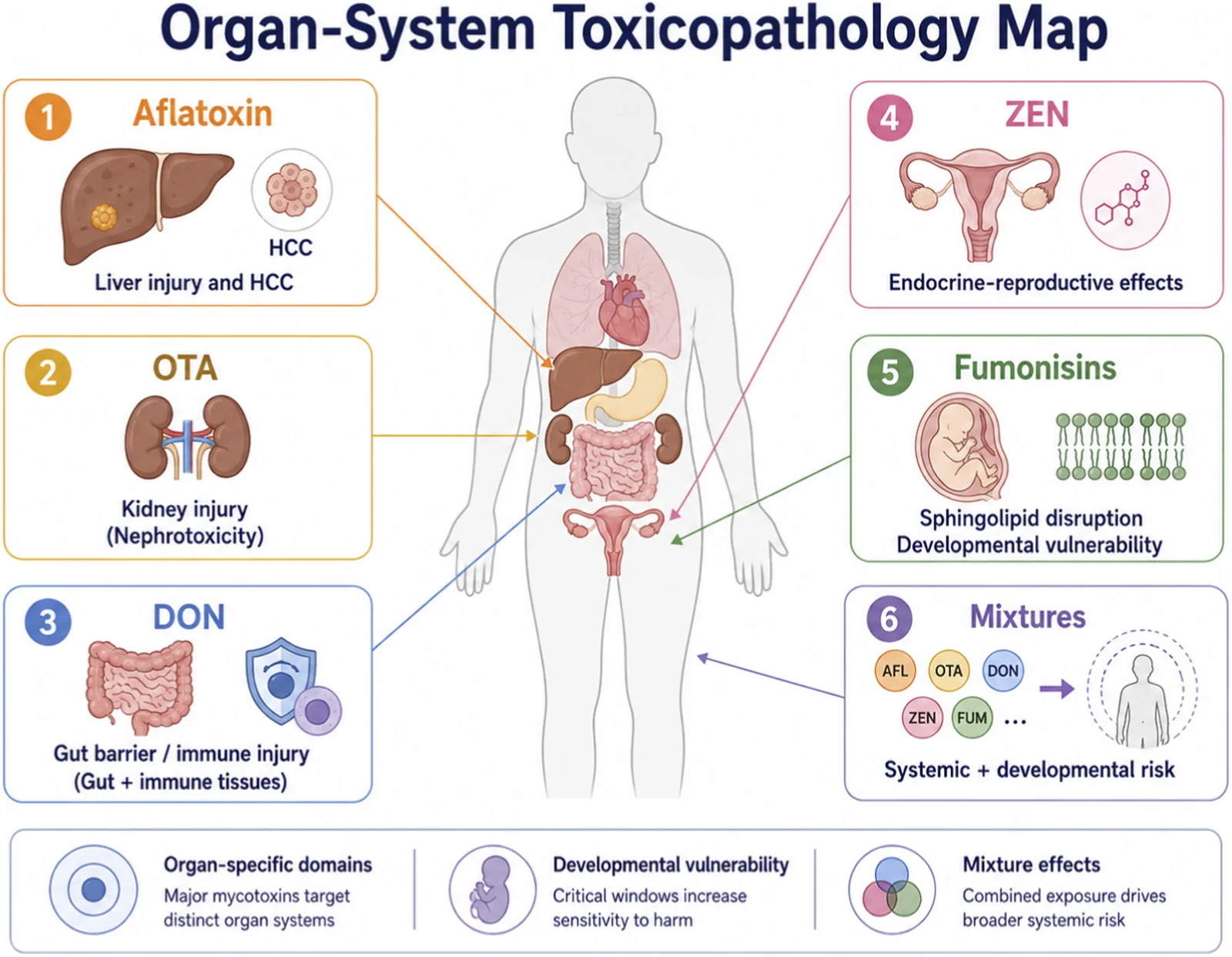
Organ-system toxicopathology map. The map presents an evidence gradient: aflatoxin has the strongest human evidence for liver injury and HCC; OTA, DON, ZEN, and fumonisins have important but less complete human evidence in kidney, gut/immune, endocrine-reproductive, and developmental domains; mixture effects remain a major research gap (National Toxicology Program, 2021; IARC, 2002; IARC, 1993; Pinton and Oswald, 2014; Marasas et al., 2004).

Table 5 summarizes this evidence hierarchy, and Figure 5 provides a visual overview. Neither should be interpreted as evidence that all depicted crop-to-organ pathways have been demonstrated in one population.

**TABLE 5:** Organ-system toxicopathology evidence map for major mycotoxin groups (IARC, 2002; IARC, 1993; National Toxicology Program, 2021; Pinton and Oswald, 2014; Marasas et al., 2004).

System	Toxin(s)	Principal biological domain	Human evidence	Priority gap
Liver	Aflatoxin B1	DNA adducts, oxidative stress, p53 mutation and HBV synergy; AFB1-lysine and AFM1 markers	Strongest human evidence: Aflatoxin-HCC	Climate-linked exposure cohorts
Kidney	OTA	Proximal tubular, oxidative and mitochondrial injury; OTA and renal markers	Strong experimental evidence; limited causal human evidence	Longitudinal human studies
Immune system	Aflatoxins, trichothecenes, OTA	Immune suppression, cytokine disruption and barrier effects	Mainly mechanistic and animal evidence; limited causal human evidence	Integrated exposure-immunity studies
Endocrine/reproductive	ZEN, mixtures	Estrogen-receptor and reproductive effects; ZEN metabolites	Strong animal evidence; limited human data	Prospective endocrine and reproductive studies
Developmental	Fumonisins, mixtures	Sphingolipid disruption and birth outcomes	Mechanistic and animal evidence with epidemiological signals; limited causal evidence	Maternal-child cohorts
Gut	DON, trichothecenes	Epithelial and barrier injury, vomiting and inflammation	Mechanistic and animal evidence; limited human dose-response data	Microbiome-toxicology integration
Cancer beyond liver	OTA, fumonisins, mixtures	Genotoxic, oxidative and proliferative pathways	Limited or inconsistent human evidence	Registry-linked prospective studies

Source: Evidence strength is deliberately conservative and separates strong aflatoxin-HCC, evidence from mechanistic, animal, or limited human evidence for other toxins (IARC, 2002; IARC, 1993; National Toxicology Program, 2021; Pinton and Oswald, 2014; Marasas et al., 2004).

## 11 AI-supported mycotoxin prediction: current evidence and realistic next steps

AI can assist because mycotoxin risk reflects nonlinear interactions among weather, crop stage, soil, injury, fungal ecology, toxin-gene potential, storage, consumption, and vulnerability. Existing models mainly predict toxin occurrence or threshold exceedance; the proposed extension is staged prediction across the crop-to-exposure pathway, with each step anchored to measured data (Focker et al., 2025). Representative predictive models and their principal limitations are compared in Table 6.

**TABLE 6:** AI and predictive-modelling benchmark table for pre-harvest mycotoxin risk prediction (Focker et al., 2025; Branstad-Spates et al., 2023; Gachoki et al., 2025).

Study/model	Inputs	Outcome	Reported result	Main limit	Authors’ appraisal
Battilani et al. Europe model	Climate scenarios, maize/wheat, spatial grid	Aflatoxin B1 risk	Mean maize AFI rose from 38 to 73 under +2 °C and 95 under +5 °C	Climate-risk model, not mycobiome-integrated	Spatial prioritization only; local calibration and toxin confirmation remain necessary
Chauhan et al. APSIM model	Crop phenology, temperature, drought index	Aflatoxin contamination	11.5/32.5/42.5 °C; drought index ≤ 0.2	Requires field calibration	Mechanistic features should inform AI
Branstad-Spates et al. Iowa GBM	Historical aflatoxin, weather, satellite, soil	Aflatoxin risk thresholds	Accuracy 96.77% (20 ppb) and 90.32% (5 ppb); lower balanced accuracy	Class imbalance	AI papers must report balanced accuracy and validation
Gachoki et al. East Africa ML	907 pre-harvest maize samples, climate, soil, topography	Low/medium/high aflatoxin risk	Balanced accuracy 62%; weak medium/high-risk validation	Sparse high-risk samples	LMIC models need better high-risk sampling
Qi et al. Wheat RF	Environment + harvest/storage wheat mycobiome	DON contamination	Reported accuracy >90%	Retrospective; no external geographic validation	Shows added value of biological indicators; prospective validation is needed
Stutt et al. Farm-to-factory model	Pre-harvest and post-harvest A. flavus/AFB1 dynamics	Supply-chain contamination	Links pre- and post-harvest risk	Limited by data availability	Risk must be modelled as a continuum
Proposed SMRI model	Climate, crop, mycobiome, toxin-gene, antagonist and injury data	Prospective field toxin risk; biomarkers are downstream validation	No performance data; prospective validation required	Unvalidated score; leakage and transferability risks	Retain only if it improves calibration, discrimination, and lead time over simpler baselines

Source: Performance values are study-specific and not directly comparable because outcomes, thresholds, prevalence, sampling, and validation differ. Balanced accuracy, calibration, external validation, and false-negative reporting are essential.

### 11.1 Prediction target: staged transitions

The primary target should remain a defined, measurable event: crop mycobiome change, toxigenic activation, toxin concentration, post-harvest amplification, or biomarker positivity. Separate stage-specific models are more defensible than one end-to-end model when intermediate data are missing. Each stage also has a different intervention, from field sampling and biocontrol to drying, segregation, biomonitoring, or clinical follow-up.

#### 11.1.1 Temporal order is essential

Drought before flowering, water stress during grain filling, rewetting, delayed harvest, and humid storage have different meanings. Models should encode sequence, duration, phenology, and sampling time rather than treating all measurements as simultaneous covariates (Chauhan et al., 2015; Stutt et al., 2023).

Mechanistic crop-fungus models, tree-based machine learning, geospatial and Bayesian models, and network-aware approaches answer different questions. Choice should follow the outcome, sample size, data structure, and need for uncertainty or biological interpretation rather than novelty of the algorithm (Focker et al., 2025).

### 11.2 Hybrid model ecosystem

A practical system should combine mechanistic and data-driven models. Mechanistic outputs can encode crop phenology, fungal thermal biology, and water stress; machine learning can then capture residual nonlinearities and spatial heterogeneity. This is more interpretable than a black-box model and more flexible than a mechanistic model used without local calibration (Chauhan et al., 2015; Camardo Leggieri et al., 2021).

Tree-based classifiers can integrate weather, soil, satellite, crop, fungal, and management variables, but class imbalance is a recurrent limitation. Overall accuracy should be reported with class-specific sensitivity, specificity, balanced accuracy, calibration, and external validation, because rare high-risk events are the most important to detect (Branstad-Spates et al., 2023; Gachoki et al., 2025).

Geospatial models can prioritize sampling and storage surveillance, whereas Bayesian hierarchical models can represent uncertainty and uneven regional sampling. Both require local calibration before transfer across crops, climates, cultivars, or supply chains (Battilani et al., 2016; Gachoki et al., 2025).

Network-aware models are relevant only when the underlying network is credible. Co-occurrence edges can generate hypotheses about community structure, but they do not establish competition or causality. Network features should be retained only if they improve externally validated prediction beyond abundance and environmental baselines.

Explainability should support, not replace, validation. Feature importance, partial dependence, or SHAP analyses can identify the variables driving a prediction, but a plausible explanation does not prove a causal mechanism (Lundberg and Lee, 2017; Molnar, 2022).

Digital twins remain a future research architecture. Their value would lie in updating a crop-to-food risk model as weather, field, storage, and assay data arrive; current evidence does not support routine crop-to-human digital twins.

Every sample should retain field or lot identity, date, crop stage, weather window, cultivar where available, crop injury, storage trajectory, assay method, limit of detection, limit of quantification, and downstream use. Without these identifiers, models may learn region or sampling artefacts rather than biology (Focker et al., 2025).

The minimum biological dataset should pair mycobiome profiling with absolute or semi-quantitative fungal measures and chemical toxin assays. Relative abundance can rise when other taxa fall, and toxin-gene presence does not establish expression or toxin accumulation. Storage and dietary variables are required only when the prediction target extends beyond the field (Tedersoo et al., 2022; Lanubile et al., 2021).

Active learning may help allocate expensive sequencing or LC-MS/MS assays to uncertain or high-risk lots, but it should first be evaluated prospectively against fixed sampling strategies. Its benefit is efficient information gain, not automatic risk control.

Counterfactual or scenario outputs should be labelled cautiously. A model may estimate that rapid drying or additional sampling changes predicted risk, but observational predictions do not establish intervention effects. Controlled or quasi-experimental data are needed before presenting a modelled action as causal.

Current studies illustrate the available evidence. Battilani et al. (2016) mapped climate-linked aflatoxin suitability; Chauhan et al. (2015) incorporated crop water stress and fungal temperature thresholds; Branstad-Spates et al. (2023) combined historical contamination, weather, satellite, and soil data; Castano-Duque et al. (2025) combined mechanistic indices with remote-sensing, soil, and meteorological features for Texas maize; Gachoki et al. (2025) tested maize risk classes across East and Southern Africa; Stutt et al. (2023) linked pre- and post-harvest stages; and Qi et al. (2024) combined environmental features and wheat mycobiome indicators to predict DON contamination. These studies cover important adjacent stages but not the full field-to-human axis.

### 11.3 Lessons from current predictive studies

Branstad-Spates et al. (2023) reported high overall accuracy for Iowa maize at 5- and 20-ppb thresholds, but lower balanced accuracy exposed the effect of rare positive cases. The study is valuable both for its multimodal inputs and for showing why average accuracy alone is insufficient.

Gachoki et al. (2025) analysed 907 pre-harvest maize samples and found that gradient boosting improved balanced accuracy, yet external validation remained weak for medium- and high-risk classes. The limiting factor was not only algorithm choice but sparse representation of the events the model needed to detect.

Qi et al. (2024) sampled wheat during harvest and storage across 12 Chinese provinces. Contaminated and uncontaminated lots differed in mycobiome composition and co-occurrence structure; Fusarium was the principal differential taxon, and random-forest models using biological and environmental indicators achieved reported accuracies above 90%. This is a useful mycobiome-integrated example, but independent geographic validation and prospective testing are still needed. Taken together, current studies favour hybrid, calibrated, and supply-chain-aware models over isolated black-box classifiers.

The proposed extension is a staged One Health prediction stack linking climate and crop stress to mycobiome, toxigenic potential, chemical assays, storage, consumption, and biomonitoring. It is a research design rather than a currently deployable model; human biomarkers should be downstream validation outcomes, not pre-harvest predictors.

### 11.4 One health prediction stack

Layer 1 combines heat, rainfall sequence, drought, soil moisture, crop phenology, satellite stress indicators, and injury. These variables describe environmental pressure and host vulnerability and can be collected at field scale (Battilani et al., 2016; Chauhan et al., 2015).

Layer 2 adds mycobiome composition, absolute toxigenic fungal abundance, antagonist taxa, toxigenic strain or gene measures, and chemical toxin confirmation. Fungal detection, gene potential, expression, and toxin accumulation remain separate endpoints (Tedersoo et al., 2022; Lanubile et al., 2021).

Layer 3 adds drying, storage, transport, processing, dietary use, and human biomonitoring when the goal is exposure prediction. These data should not be folded into a pre-harvest score because doing so would obscure where risk arose and could introduce predictor-outcome leakage (Stutt et al., 2023; Owolabi et al., 2024).

Outputs should be stage-specific probabilities with uncertainty: dysbiosis, toxigenic activation, toxin occurrence, storage amplification, biomarker positivity, and intervention priority. A single label would hide whether the actionable problem is in the field, storage system, or exposed population.

A risk trajectory also prevents false reassurance. High climate risk may not progress when suppression remains intact, whereas moderate field risk may become important after drying failure or in a staple-dependent population. The model should preserve these distinct pathways.

Each output must be tied to an authorized action. Only representative chemical analysis can determine compliance with a legal maximum; model outputs may instead trigger sampling, drying, storage correction, biocontrol, or public-health investigation (European Commission, 2023; Stutt et al., 2023).

### 11.5 Validation and implementation principles

Validation should report class-specific sensitivity, specificity, balanced accuracy, calibration, uncertainty, threshold-dependent performance, and external testing across seasons and regions. Risk-stratified sampling and cost-sensitive methods may address class imbalance, but false negatives must remain visible (Branstad-Spates et al., 2023; Gachoki et al., 2025).

Transferability cannot be assumed. Models should be locally calibrated and tested across crops, cultivars, climates, storage systems, and dietary contexts. In low-resource settings, their near-term role is triage of limited laboratory, drying, storage, or biomonitoring capacity (Focker et al., 2025).

Data governance is an implementation safeguard, not a substitute for model validity. Access, uncertainty communication, farm-data ownership, consent for human data, and protection against unsupported regional or farmer-level penalties should be specified before deployment.

A feasible tiered system begins with climate, soil, satellite, and regional-history screening; adds field inspection, storage data, and rapid toxin tests in selected locations; and reserves sequencing, toxin-gene assays, LC-MS/MS, or biomonitoring for sentinel or high-uncertainty settings.

Causal diagrams should distinguish upstream drivers, mediators, and downstream outcomes before model fitting. Otherwise, storage or toxin variables may mask the value of a pre-harvest signal, and feature importance may be misread as mechanism.

Temporal alignment is equally important: satellite data, field mycobiome sampling, storage assays, and urine biomarkers represent different biological windows. Dates, crop stage, storage duration, and biomarker timing should be harmonized before multimodal fusion.

Operational outputs should show the risk stage, uncertainty, main drivers, missing data, and recommended next measurement or action. Scenario comparisons should be clearly separated from proven intervention effects.

**FIGURE 6:**
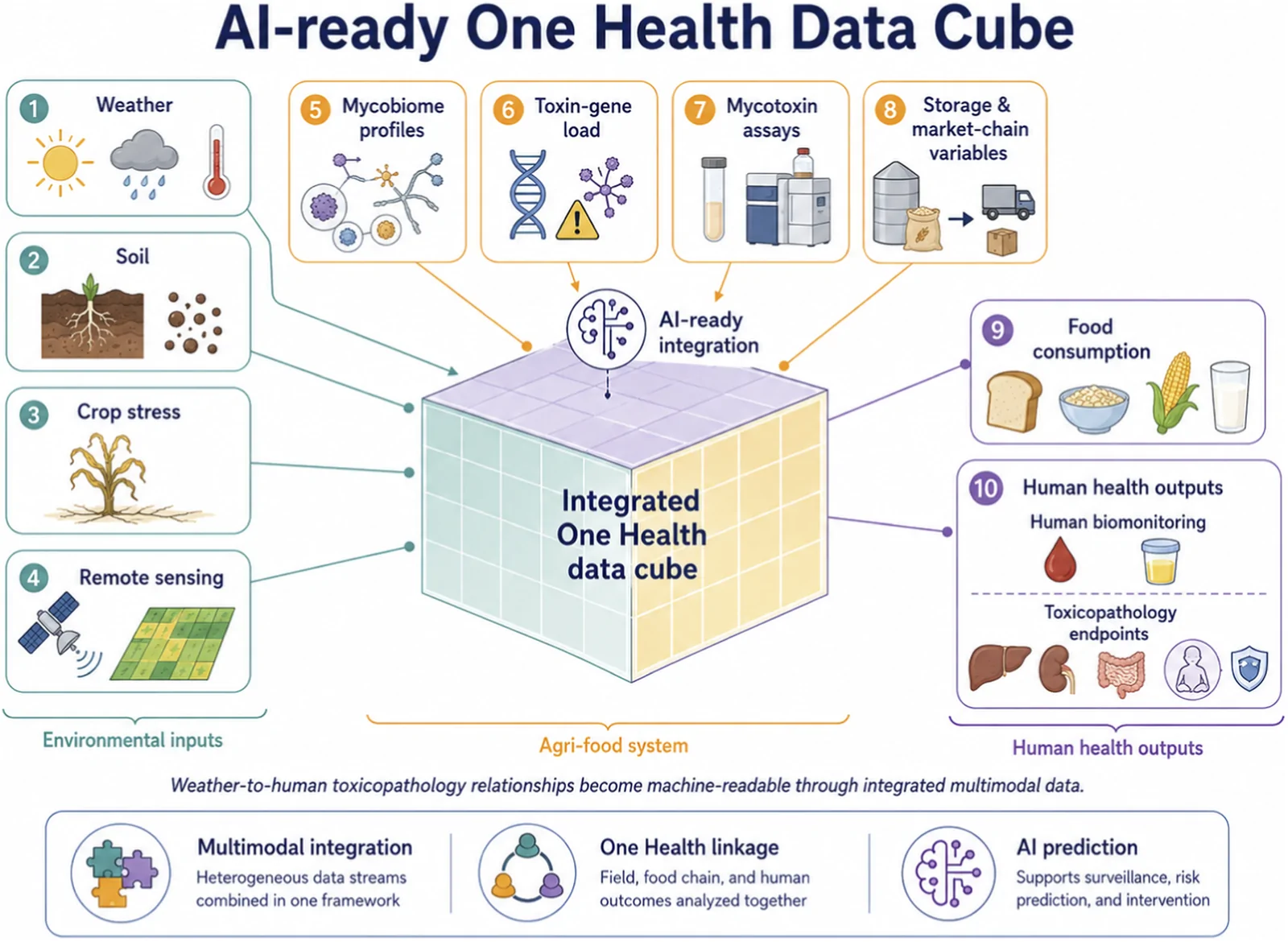
Proposed AI-ready One Health data architecture. The diagram lists environmental, crop, mycobiome, toxin-gene, assay, storage, consumption, biomonitoring, and toxicopathology data needed to test staged prediction. It is a research design, not a validated or routinely available data cube (Focker et al., 2025; Stutt et al., 2023; Owolabi et al., 2024).

Figure 6 summarizes the minimum data architecture for testing staged prediction across field, molecular, chemical, food-chain, and human-exposure layers.

## 12 Proposed sentinel mycobiome risk index

The Sentinel Mycobiome Risk Index (SMRI) is proposed as a research hypothesis for estimating whether a crop system is moving toward toxigenic contamination. It is not a validated agricultural, regulatory, or clinical score and must not replace representative sampling and chemical analysis. Its proposed use is to test whether a small set of ecological variables can prioritize where confirmatory testing, drying, storage control, or intervention should be evaluated (Battilani et al., 2016; Katati et al., 2023; Focker et al., 2025).

A core pre-harvest SMRI could combine heat anomaly, drought stress, rainfall instability, absolute or semi-quantitative toxigenic fungal abundance, toxigenic strain or gene measures, antagonist depletion, and crop injury. Storage moisture, drying delay, and storage temperature should be analysed as a separately labelled post-harvest extension because they occur after the proposed field-sentinel stage. Human biomarkers are downstream validation outcomes and must never be included as score components. All weights should be crop-, region-, stage-, and toxin-specific. Table 7 provides the revised data dictionary (Chauhan et al., 2015; Katati et al., 2023; Agbetiameh et al., 2020; Stutt et al., 2023).

**TABLE 7:** Proposed Sentinel Mycobiome Risk Index data dictionary (Battilani et al., 2016; Chauhan et al., 2015; Katati et al., 2023; Agbetiameh et al., 2020).

SMRI feature	Measurement	Risk direction	Anchor	Evidence status/use
Heat anomaly	Growing-season temperature deviation	Higher heat increases aflatoxin risk in susceptible systems	A. flavus optimum near 32.5 °C in APSIM model	Captures fungal thermal suitability
Drought stress	Soil moisture, crop water supply:demand ratio	Higher drought increases risk	<=0.2 crop water supply:demand ratio as severe stress marker	Captures host stress and kernel vulnerability
Rainfall instability	Dry spell followed by humidity/rain	Higher instability increases risk	Zambia dry-spell mycobiome shifts	Captures compound stress
Toxigenic fungal abundance	ITS/qPCR/metagenomics	Higher abundance increases risk	Aspergillus/Fusarium relative abundance linked to toxins in Zambia	Captures community-level warning
Toxin gene load	aflR, aflD, fum1, tri5, pks genes	Higher gene load increases risk	To be extracted across studies	Moves beyond genus-level identification
Toxigenic network centrality	Co-occurrence network metrics	Higher centrality increases risk	Author-proposed feature; no validated cut-point	Identifies toxigenic fungi as ecological hubs
Antagonist depletion	Atoxigenic Aspergillus, Trichoderma, *Bacillus*, yeasts	Lower antagonist abundance increases risk	Atoxigenic biocontrol reduced aflatoxin up to 76%–100% in Ghana	Quantifies suppression debt
Crop injury	Insect damage, drought injury, lodging	Higher injury increases risk	Insect-derived ear injury is linked with A. flavus infection and aflatoxin accumulation in maize	Direct crop-injury anchor; context varies by insect, hybrid, climate, and management
Storage amplification	Moisture, drying delay, storage temperature	Higher amplification increases risk	Stutt et al. Farm-to-factory modelling	Post-harvest extension, not a core pre-harvest field-score component
Downstream validation outcome (not an SMRI component)	Urinary, blood, or breast-milk biomarkers in linked studies	Used to test translation; excluded from pre-harvest score calculation	DON was quantified above LOQ in 99% of one German 24-h urine series	Prevents predictor-outcome leakage and tests whether crop risk reaches internal exposure

Source: SMRI, is an author-proposed research construct. No validated weights, cut-points, or food-release decisions currently exist. Core pre-harvest variables, post-harvest extensions, and downstream validation outcomes must be analysed separately and standardized by crop, region, sampling stage, and analytical platform (Battilani et al., 2016; Chauhan et al., 2015; Katati et al., 2023; Agbetiameh et al., 2020; Pruter et al., 2020; Riungu et al., 2024; Stutt et al., 2023).

The potential value of SMRI is empirical rather than rhetorical: it should be retained only if it predicts measured toxin occurrence or intervention response better than simpler climate-only and crop-injury models. A score that includes many expensive variables but adds little discrimination, calibration, or lead time would not be useful. The index should therefore be compared with current testing strategies and reported with transparent component contributions and uncertainty (Focker et al., 2025; Katati et al., 2023).

A research-stage SMRI should be transparent enough for prospective validation and modular enough to test low-cost and high-resolution versions. Components must be standardized within crop, region, and sampling stage and calibrated separately for maize-aflatoxin, wheat-DON/ZEN, groundnut-aflatoxin, and storage-linked OTA systems. This crop-specific design avoids a one-size-fits-all score and allows investigators to determine whether sequencing or network features add value over weather, crop injury, storage moisture, and rapid toxin screening (Chauhan et al., 2015; Giorni et al., 2019; Katati et al., 2023).

The index should distinguish pre-harvest risk drivers from protective features and from post-harvest extensions. Toxigenic fungal abundance, toxigenic strain or gene measures, crop injury, prior regional contamination, and author-proposed network centrality would increase the core score; antagonist abundance, atoxigenic competitor dominance, and proven suppressive management would reduce it. Drying delay, storage moisture, and rapid drying should be reported in a separate post-harvest extension. This separation gives the index mechanistic meaning and prevents later food-chain information from leaking into an apparently pre-harvest score (Agbetiameh et al., 2019; Banerjee et al., 2018; Xiong et al., 2021; Stutt et al., 2023).

Risk tiers are presented only as a future validation scenario. Before any operational use, cut-points would require prospective calibration, external validation, cost-effectiveness analysis, and comparison with representative toxin testing. A validated low-risk tier might support routine monitoring, whereas higher predicted risk might justify additional sampling or preventive drying and storage actions; no tier should authorize food release, rejection, biomonitoring, or clinical follow-up on the score alone (European Commission, 2023; Stutt et al., 2023; Owolabi et al., 2024).

SMRI validation should proceed in stages. First, studies should test whether the core pre-harvest score predicts toxin occurrence better than climate variables alone. Second, they should test whether adding mycobiome network features improves prediction beyond toxigenic abundance. Third, intervention studies should test whether interventions that reduce the relevant pre-harvest or separately reported post-harvest component scores also reduce final toxin levels. Fourth, paired field-food-human studies should test whether the pre-harvest score predicts downstream biomarker positivity or mixture burden without incorporating those outcomes into the score. This sequence would turn the index from a conceptual proposal into a measurable systems-biology tool (Agbetiameh et al., 2020; Katati et al., 2023; Schmied et al., 2023).

SMRI should be reported with uncertainty rather than as a deterministic number. Uncertainty can arise from missing climate data, uneven field sampling, relative-abundance sequencing, unmeasured storage conditions, toxin analytical limits, or unknown dietary pathways. Reporting an uncertainty band would prevent overconfidence and would help distinguish fields with lower predicted risk from fields that are data-poor. This is particularly important when the index is used to prioritize sampling or intervention under limited resources (Focker et al., 2025; Branstad-Spates et al., 2023; Gachoki et al., 2025).

An additional design correction is required: human biomarkers are outcomes for validating downstream translation and should not contribute to a pre-harvest field score. Storage variables may be reported either as a separate post-harvest extension or as a clearly labelled second-stage score. Keeping predictors and validation outcomes separate will reduce leakage and circular model performance.

The index could bridge low-cost field scoring and high-resolution research only if its stages remain separable. Candidate core pre-harvest predictors include heat, drought, rainfall sequence, crop injury, historical contamination, and low-cost fungal or toxin-potential measures where available. A separately labelled post-harvest extension could add drying delay, storage moisture, and storage temperature. Research versions may add absolute fungal measures, toxigenic strain identity, gene expression, antagonist activity, and network features. Rapid toxin screening is a confirmatory or comparison measurement rather than a pre-harvest ecological predictor, and human biomarkers are downstream validation outcomes (Focker et al., 2025; Cramer et al., 2026).

## 13 Methodological challenges and research roadmap

Several limitations must be addressed before the axis becomes operational. First, colonization is not toxin production. Relative abundance from ITS sequencing can indicate fungal-community structure, but it cannot reliably distinguish toxigenic from atoxigenic strains or gene expression. qPCR, metagenomics, targeted toxin-gene assays, transcriptomics, and direct toxin quantification should be paired where possible. Second, sequencing has biases related to primer choice, copy-number variation, relative abundance, taxonomic resolution, sample timing, and lack of absolute abundance. Spike-ins, absolute qPCR, culture-based confirmation, and toxin assays can reduce overinterpretation (Tedersoo et al., 2022; Giorni et al., 2019; Lanubile et al., 2021).

Third, AI studies face class imbalance, non-transferability, missing LMIC data, inconsistent toxin thresholds, and limited external validation. High overall accuracy can be misleading when high-risk contamination events are rare, so balanced accuracy, sensitivity, specificity, calibration, threshold-dependent performance, external validation, and interpretability are required (Branstad-Spates et al., 2023; Gachoki et al., 2025; Focker et al., 2025). Fourth, human biomonitoring has limitations related to half-life, matrix choice, timing, dietary recall, co-exposure complexity, and lack of paired crop-human data (Owolabi et al., 2024; Cramer et al., 2026).

Fifth, intervention evidence should be matched to the stage at which the intervention acts. A field biocontrol intervention should first be judged by ecological and commodity-toxin outcomes; a drying or storage intervention by post-harvest amplification and toxin outcomes; and a population intervention by exposure reduction. For interventions intended to interrupt several stages, downstream endpoints may include toxigenic dominance, suppressive network structure, toxin-gene activity, commodity toxin burden, and human biomarkers. No single intervention should be considered unsuccessful merely because it was not designed or powered to measure every downstream stage (Agbetiameh et al., 2019; Agbetiameh et al., 2020; Stutt et al., 2023; Owolabi et al., 2024).

Sixth, future datasets must be interoperable. Climate records, satellite products, sequencing data, toxin assays, storage measurements, dietary surveys, biomarker panels, and clinical endpoints often use different identifiers, time scales, and units. Without shared metadata, harmonized sampling dates, geolocation, crop stage, analytical limits, and matrix-specific biomarker interpretation, AI models will remain difficult to validate. Data architecture is therefore not a technical afterthought; it is a scientific prerequisite for testing the Crop Mycobiome-Exposome-Toxicopathology Axis (Focker et al., 2025; Owolabi et al., 2024; Cramer et al., 2026).

Table 8 translates these gaps into testable hypotheses. The most urgent studies are paired field-food-human cohorts that measure climate stress, crop mycobiome structure, toxin-gene potential, actual toxin concentrations, storage conditions, diet, urine and blood biomarkers, and organ-system endpoints. Intervention studies should test whether atoxigenic biocontrol, synthetic suppressive consortia, improved drying, storage control, or risk-based sorting reduce not only crop toxin concentration but also human biomarkers such as AFB1-lysine, urinary AFM1, or urinary DON (Agbetiameh et al., 2020; Owolabi et al., 2024; Schmied et al., 2023; Sun et al., 1999).

**TABLE 8:** Data-generating hypotheses for future One Health studies (Katati et al., 2023; Stutt et al., 2023; Owolabi et al., 2024).

Hypothesis	Minimum dataset	Primary endpoint	Decision rule/evidence needed
SMRI predicts toxin contamination earlier than toxin testing	Climate + crop mycobiome + toxin assay	Time from dysbiosis signal to toxin exceedance	Requires lead time and improvement over climate-only and crop-injury baselines
Antagonist depletion predicts aflatoxin independent of Aspergillus abundance	Mycobiome + antagonist qPCR + aflatoxin	Aflatoxin concentration	Tests microbiome suppression debt
Climate stress changes toxin mixtures, not only toxin quantity	Multi-toxin LC-MS/MS + weather	Mixture burden score	Moves beyond single-toxin risk
Pre-harvest SMRI predicts human urinary biomarkers	Field mycobiome + food toxin + urine/blood biomarkers	Biomarker concentration	Connects crop ecology to human exposome
Atoxigenic biocontrol lowers human biomarkers	Intervention + crop toxin + human biomonitoring	Change in AFB1-lysine or AFM1	Requires paired intervention, crop-toxin, and biomarker outcomes; currently unproven
Explainable AI identifies biologically meaningful warning signals	Multimodal dataset + SHAP/feature importance	Model interpretability	Builds trust and mechanistic plausibility
Category-specific legal limits should not be interpreted as biological-effect ratios	Current regulation text + matched food categories + processing stage + consumption/exposure data	Category-specific exposure estimate	Compare matched categories and model intake; reject simple ratios between unlike foods as effect sizes

Source: These hypotheses are intended to convert the conceptual framework into testable field, intervention, and biomonitoring designs (Katati et al., 2023; Stutt et al., 2023; Owolabi et al., 2024).

A useful roadmap should prioritize studies that connect at least three adjacent stages of the axis. Climate-to-toxin studies are valuable, but climate-to-mycobiome-to-toxin studies are stronger. Toxin-to-biomarker studies are valuable, but crop-to-food-to-biomarker studies are stronger. The highest-value designs would follow a crop from climate exposure through field mycobiome sampling, toxin-gene measurement, harvest toxin analysis, storage tracking, household food use, human biomarkers, and early organ-system endpoints. Such studies would directly test whether the proposed sentinel state predicts human exposure (Katati et al., 2023; Owolabi et al., 2024; Stutt et al., 2023).

Intervention studies should use the same logic. If atoxigenic Aspergillus, suppressive consortia, improved drying, storage modification, or risk-based sorting reduce final toxin levels, the next question is whether they also reduce biomarker burden in exposed communities. This crop-to-human endpoint would transform mycotoxin intervention evidence from agricultural efficacy to One Health prevention (Agbetiameh et al., 2020; Stutt et al., 2023; Owolabi et al., 2024).

## 14 Discussion, evidence appraisal, and conclusions

The evidence supports a linked but incomplete sequence from climate and crop stress to fungal ecology, measured toxin, food-chain handling, human exposure, and organ effects. The Crop Mycobiome-Exposome-Toxicopathology Axis is therefore best viewed as an evidence map that identifies adjacent links and missing tests, not as proof that every field progresses through a fixed ecological-to-clinical pathway. AI can combine measured data across stages, but it cannot supply causality or fill unmeasured links (Stutt et al., 2023; Focker et al., 2025; Owolabi et al., 2024).

Five conclusions are well supported. First, climate and crop water stress can alter aflatoxin suitability in defined crop-region systems. Second, pre-harvest maize fungal-community composition is associated with aflatoxin and fumonisin occurrence in field studies. Third, atoxigenic Aspergillus biocontrol can substantially reduce aflatoxin in maize and groundnut. Fourth, human biomonitoring can quantify internal exposure. Fifth, aflatoxin has a strong mechanistic and epidemiological link with hepatocellular carcinoma. The strength, scale, and directness of evidence are not equal across these links (Battilani et al., 2016; Katati et al., 2023; Agbetiameh et al., 2020; Schmied et al., 2023; Owolabi et al., 2024; IARC, 2002; National Toxicology Program, 2021; Sun et al., 1999).

The practical conclusion is not that control should move exclusively upstream. Effective prevention requires a continuum: climate-informed field management and targeted sampling before harvest; representative toxin testing at harvest and market stages; rapid drying, insect control, traceability, sorting, and safe storage after harvest; and exposure assessment where public-health concern remains. Earlier ecological information is useful only if it improves the timing or targeting of these established measures (World Health Organization, 2023a; European Commission, 2023; Karlovsky et al., 2016; Pascale et al., 2020; Stutt et al., 2023).

Important uncertainties remain. Dysbiosis is not synonymous with low diversity; co-occurrence networks do not prove interaction; fungal detection does not establish toxigenicity; a non-detect does not establish chronic dose; and regulatory maximum levels cannot be converted into biological-risk ratios by comparing digits alone. Post-harvest conditions can amplify or override pre-harvest signals, and processing can redistribute toxins into fractions rather than remove them completely. These boundaries limit the claims that can presently be made (Tedersoo et al., 2022; European Commission, 2023; Karlovsky et al., 2016; Pascale et al., 2020).

The authors’ contribution is to make these evidence boundaries explicit and to convert them into comparative questions. Does a mycobiome measure add predictive value beyond weather and crop injury? Does antagonist loss predict toxin after adjustment for fungal biomass? Do pre-harvest signals remain informative after storage conditions are included? Does an intervention reduce human biomarkers as well as grain toxin? These questions generate testable synthesis rather than treating adjacent literatures as proof of a completed axis.

Three elements remain proposals: microbiome suppression debt, toxigenic network centrality as a risk feature, and SMRI. Their validation should proceed in sequence: establish reproducible measurement; demonstrate temporality; show external predictive improvement over simpler models; test whether score-guided action changes toxin outcomes; and only then evaluate downstream biomarker or health relevance. Negative findings would be informative because they would define where climate, storage, or direct toxin testing is sufficient.

Paired prospective studies provide the decisive test. Fields with similar climate stress but different antagonist abundance or toxigenic activity should be followed through harvest, drying, storage, household use, and human biomarkers. Analyses should include negative controls, absolute fungal measures, limits of detection and quantification, processing factors, consumption data, and pre-specified evidence thresholds. Without this design, the full axis will remain a plausible integration of separate literatures rather than a validated One Health pathway (Katati et al., 2023; Stutt et al., 2023; Owolabi et al., 2024).

Interventions should be evaluated at the stage they are intended to change. Biocontrol should first demonstrate reproducible reduction in toxigenic fungi and grain toxin; drying and storage interventions should reduce post-harvest amplification; exposure interventions should reduce dietary intake or biomarkers. Demonstrating effects across several stages would strengthen One Health translation, but failure to measure a downstream endpoint should not erase a well-established agricultural benefit (Agbetiameh et al., 2020; Stutt et al., 2023; Owolabi et al., 2024).

In conclusion, crop mycobiome data should be judged by a practical standard: whether they improve prediction, sampling, or intervention beyond weather, crop inspection, storage monitoring, and chemical toxin analysis. Current evidence is sufficient to justify prospective testing of this added value, but not to claim that the full axis or SMRI is validated. The most defensible direction is prevention across the entire crop-to-food continuum, with earlier ecological warning linked to, rather than substituted for, post-harvest control and human exposure assessment.
