# Supplementary material for: Climate-stressed crop mycobiomes as pre-harvest sentinels of human mycotoxin exposure: an AI-enabled One Health framework linking dysbiosis, exposomics, and toxicopathology
Source: Front Syst Biol. 2026 Aug 28;6:1921729. doi: 10.3389/fsysb.2026.1921729 (PMC13563512; doi:10.3389/fsysb.2026.1921729)
Supplement: Supplementary file 1 [file Table1.docx]

**Table 1.** Climate-mycobiome-toxin transition thresholds relevant to pre-harvest sentinel surveillance (Battilani et al., 2016; Chauhan et al., 2015; Katati et al., 2023).

| **Risk layer** | **Data/value to extract** | **Biological meaning** | **Key source** | **Novel insight** |
| --- | --- | --- | --- | --- |
| European maize aflatoxin risk | Mean aflatoxin risk index: present 38; +2 °C 73; +5 °C 95 | Warming expands aflatoxin-suitable environments | Battilani et al., 2016 | Climate warming can be translated into pre-harvest toxin-risk maps |
| High-risk European maize sites | Sites above high-risk threshold: 20% present; 39% at +2 °C; 54% at +5 °C | Risk becomes geographically broader | Battilani et al., 2016 | Northward or regional risk shifts should guide sampling |
| A. flavus thermal niche | Growth 30-35 °C; aflatoxin production 25-35 °C at high water activity | Hot conditions favour aflatoxin systems | Giorni et al., 2019 | Growth and toxin biosynthesis overlap but are not identical |
| F. graminearum niche | Growth 15-25 °C; DON production 15-25 °C | Cooler and humid systems favour DON risk | Giorni et al., 2019 | Climate change may reshuffle toxin dominance |
| Aflatoxin mechanistic model | A. flavus base/optimum/maximum temperatures 11.5/32.5/42.5 °C; drought index <=0.2 | Combines crop stress and fungal biology | Chauhan et al., 2015 | Biologically interpretable thresholds should inform AI features |
| Zambia maize mycobiome | 40 fields; 61 fungal genera; dry spell linked with Aspergillus and Fusarium patterns | Rainfall pattern restructures pre-harvest mycobiome | Katati et al., 2023 | Pre-harvest mycobiome is a measurable sentinel |
| Regulatory versus detectable contamination | Approximately 25% exceed regulatory limits while 60-80% may show detectable contamination | Legal exceedance underestimates biological exposure | Eskola et al., 2020 | Exposure-burden metrics are needed beyond pass/fail regulation |

*Source note: Values summarize ranges from the cited studies and are synthesis anchors rather than universal thresholds (Battilani et al., 2016; Giorni et al., 2019; Chauhan et al., 2015; Katati et al., 2023).*

**Table 2**. Crop-fungus-toxin-climate matrix linking major commodities with climate-sensitive toxigenic guilds and human relevance (Bennett and Klich, 2003; Giorni et al., 2019; Wild and Gong, 2010; Bui-Klimke and Wu, 2015).

| **Crop/commodity** | **Main fungal guild** | **Major toxins** | **Climate-sensitive drivers** | **Human relevance** |
| --- | --- | --- | --- | --- |
| Maize | Aspergillus flavus; Fusarium verticillioides | Aflatoxins; fumonisins | Heat, drought, insect injury, dry-humid cycles | Liver cancer risk, fumonisin-linked sphingolipid disruption, child exposure |
| Groundnut | Aspergillus flavus/parasiticus | Aflatoxins | Drought, high temperature, soil contact, storage moisture | High dietary exposure in groundnut-consuming populations |
| Wheat/barley/oats | Fusarium graminearum/culmorum | DON, ZEN, T-2/HT-2 | Humidity, rainfall around flowering, moderate temperature | Gastrointestinal, immune, and endocrine effects |
| Coffee/dried fruits/grapes | Aspergillus/Penicillium species | OTA | Warm storage, drying conditions, humidity | Kidney toxicity and possible carcinogenicity |
| Spices | Aspergillus species | Aflatoxins, OTA | Hot climate, drying and storage failures | Chronic low-dose exposure and difficult decontamination |
| Milk | Feed-derived aflatoxin metabolism | AFM1 | Contaminated animal feed | Infant and child exposure through dairy |

*Source note: Guilds are simplified for synthesis; local species, chemotypes, and commodities vary by region (Bennett and Klich, 2003; Giorni et al., 2019; Wild and Gong, 2010; Bui-Klimke and Wu, 2015).*

**Table 3**. Regulatory threshold table with translational interpretation and vulnerability-aware reading of selected maximum levels (van Egmond et al., 2007; Vitalini et al., 2026; Eskola et al., 2020).

| **Toxin** | **Food category** | **Example EU maximum level** | **Translational interpretation** |
| --- | --- | --- | --- |
| Aflatoxin B1 | Cereals and cereal products | 2.0 µg/kg | Low threshold reflects high carcinogenic concern |
| Total aflatoxins | Cereals and cereal products | 4.0 µg/kg | Useful for compliance but not total mixture burden |
| Aflatoxin B1 | Maize/rice before sorting or treatment | 5.0 µg/kg | Sorting-stage limit recognizes contamination heterogeneity |
| Total aflatoxins | Maize/rice before sorting or treatment | 10.0 µg/kg | Pre-processing limits can exceed final food limits |
| AFM1 | Milk | 0.050 µg/kg | Important for children and dairy consumers |
| AFM1 | Infant formula | 0.025 µg/kg | Stricter limit reflects infant vulnerability; compression factor 2 |
| DON | Unprocessed cereal grains | 1000 µg/kg | Fusarium risk is crop and climate dependent |
| DON | Infant processed cereal-based foods | 150 µg/kg | Infant threshold illustrates vulnerability-adjusted regulation; compression factor 6.7 |
| Zearalenone | Unprocessed cereal grains | 100 µg/kg | Endocrine-disruption relevance |
| Zearalenone | Unprocessed maize | 350 µg/kg | Maize-specific Fusarium risk |
| Fumonisins B1+B2 | Unprocessed maize | 4000 µg/kg | High maize burden, important in staple-dependent diets |
| Fumonisins B1+B2 | Maize-based infant foods | 200 µg/kg | Large reduction reflects developmental vulnerability; compression factor 20 |
| OTA | Unprocessed cereals | 5.0 µg/kg | Kidney-relevant toxin |
| OTA | Roasted coffee | 3.0 µg/kg | Important non-cereal exposure route |
| OTA | Capsicum spices | 20 µg/kg | Spices can be concentrated exposure contributors |

*Source note: Maximum levels are illustrative regulatory examples used for translational interpretation; commodity-specific limits differ across jurisdictions and should be verified against the relevant current regulation before operational use (van Egmond et al., 2007; Vitalini et al., 2026; Eskola et al., 2020).*

**Table 4.** Human biomonitoring matrix linking toxins, biomarkers, matrices, exposure windows, and interpretation (Arce-López et al., 2020; Vidal et al., 2018; Owolabi et al., 2024; Cramer et al., 2026).

| **Toxin** | **Biomarker** | **Matrix** | **Exposure window** | **Key evidence/value** | **Interpretation** |
| --- | --- | --- | --- | --- | --- |
| Aflatoxin B1 | AFB1-lysine albumin adduct | Blood/serum/plasma | Longer-term exposure | Widely used biomarker in human studies | Strong liver cancer relevance |
| Aflatoxin | AFM1 | Urine, milk | Recent exposure | Qidong cohort: urinary AFM1 >3.6 ng/L linked with 3.3-fold HCC risk among chronic HBV-positive men | Strong crop-to-cancer translational anchor |
| DON | Total DON | Urine | Recent dietary exposure | German ESB: 99% above LOQ; median 4.3 µg/L; HBM guidance value 23 µg/L | Useful for population exposure surveillance |
| OTA | OTA, OTalpha | Serum/plasma, urine, breast milk | Longer persistence than many toxins | Detected in human blood and milk | Kidney and developmental relevance |
| Fumonisin B1 | FB1; sphinganine:sphingosine ratio | Urine/blood | Recent exposure/effect | Mechanistic link to sphingolipid disruption | Candidate effect biomarker |
| Zearalenone | alpha-ZEL, beta-ZEL, ZEN metabolites | Urine/serum | Recent exposure | Endocrine-disruption relevance | Important for reproductive and developmental studies |
| Multi-mycotoxin mixtures | LC-MS/MS panel | Urine, serum, plasma, breast milk | Variable | Modern biomonitoring increasingly uses LC-MS/MS/HRMS | Needed for exposome-level inference |

*Source note: Biomarker choice must consider toxicokinetics, matrix specificity, sample timing, analytical validation, and vulnerability group (Arce-López et al., 2020; Vidal et al., 2018; Owolabi et al., 2024; Cramer et al., 2026).*

**Table 5.** Organ-system toxicopathology evidence map for major mycotoxin groups (Liu and Wu, 2010; Bui-Klimke and Wu, 2015; Kensler et al., 2011; Pinton and Oswald, 2014; Marasas et al., 2004).

| **Organ/system** | **Main toxins** | **Mechanisms to discuss** | **Human evidence strength** | **Biomarkers/endpoints** | **Knowledge gap** |
| --- | --- | --- | --- | --- | --- |
| Liver | Aflatoxin B1 | DNA adducts, oxidative stress, p53 mutation, HBV synergy | Strongest for aflatoxin-HCC | AFB1-lysine, AFM1, liver enzymes, HCC | Need climate-linked exposure cohorts |
| Kidney | OTA | Proximal tubular injury, oxidative stress, mitochondrial injury | Moderate/limited human causal evidence | OTA, creatinine, eGFR, tubular markers | Need better longitudinal studies |
| Immune system | Aflatoxins, trichothecenes, OTA | Immune suppression, cytokine disruption, barrier effects | Moderate | Vaccine response, infection frequency, immune markers | Few integrated exposure studies |
| Endocrine/reproductive | ZEN, mixtures | Estrogen receptor activity, reproductive disruption | Strong animal evidence, limited human data | ZEN metabolites, fertility markers, puberty markers | Human endocrine studies needed |
| Nervous system/development | Fumonisins, mixtures | Sphingolipid disruption, fetal vulnerability | Suggestive | Sphinganine:sphingosine, birth outcomes | Need maternal-child cohorts |
| Gut | DON, trichothecenes | Epithelial injury, vomiting, inflammation, barrier dysfunction | Moderate | Gut permeability, inflammatory markers | Need microbiome-toxicology integration |
| Cancer beyond liver | OTA, fumonisins, mixtures | Genotoxicity, oxidative stress, proliferative signaling | Limited/inconsistent | Cancer registry linkage | Non-aflatoxin carcinogenicity remains understudied |

*Source note: Evidence strength is deliberately conservative and separates strong aflatoxin-HCC evidence from weaker or inferential links for other toxins (Liu and Wu, 2010; Bui-Klimke and Wu, 2015; Kensler et al., 2011; Pinton and Oswald, 2014; Marasas et al., 2004).*

**Table 6.** AI and predictive-modelling benchmark table for pre-harvest mycotoxin risk prediction (Focker et al., 2025; Branstad-Spates et al., 2023; Gachoki et al., 2025).

| **Study/model** | **Data inputs** | **Outcome predicted** | **Performance/value** | **Main limitation** | **Lesson for this review** |
| --- | --- | --- | --- | --- | --- |
| Battilani et al. Europe model | Climate scenarios, maize/wheat, spatial grid | Aflatoxin B1 risk | Mean maize AFI rose from 38 to 73 under +2 °C and 95 under +5 °C | Climate-risk model, not mycobiome-integrated | Climate maps can guide pre-harvest surveillance |
| Chauhan et al. APSIM model | Crop phenology, temperature, drought index | Aflatoxin contamination | Biological thresholds 11.5/32.5/42.5 °C; drought index <=0.2 | Requires field calibration | Mechanistic features should inform AI |
| Branstad-Spates et al. Iowa GBM | Historical aflatoxin, weather, satellite, soil | Aflatoxin risk thresholds | Overall accuracy 96.77% at 20 ppb and 90.32% at 5 ppb, but balanced accuracy lower | Class imbalance | AI papers must report balanced accuracy and validation |
| Gachoki et al. East Africa ML | 907 pre-harvest maize samples, climate, soil, topography | Low/medium/high aflatoxin risk | Gradient boosting balanced accuracy 62%; external validation weak for medium/high classes | Sparse high-risk samples | LMIC models need better high-risk sampling |
| Stutt et al. farm-to-factory model | Pre-harvest and post-harvest A. flavus/AFB1 dynamics | Supply-chain contamination | Integrates farm-to-factory risk | Limited by data availability | Risk must be modelled as a continuum |
| Proposed SMRI model | Climate, crop stress, mycobiome, toxin genes, antagonists, storage, biomarkers | Pre-harvest human exposure risk | Future validation target | Not yet validated | This Review’s novel contribution |

*Source note: Balanced accuracy, sensitivity, calibration, external validation, and biological interpretability should be reported alongside overall accuracy (Focker et al., 2025; Branstad-Spates et al., 2023; Gachoki et al., 2025).*

**Table 7.** Proposed Sentinel Mycobiome Risk Index data dictionary (Battilani et al., 2016; Chauhan et al., 2015; Katati et al., 2023; Agbetiameh et al., 2020).

| **SMRI component** | **Suggested measurement** | **Direction of risk** | **Example values or anchors** | **Why it matters** |
| --- | --- | --- | --- | --- |
| Heat anomaly | Growing-season temperature deviation | Higher heat increases aflatoxin risk in susceptible systems | A. flavus optimum near 32.5 °C in APSIM model | Captures fungal thermal suitability |
| Drought stress | Soil moisture, crop water supply:demand ratio | Higher drought increases risk | <=0.2 crop water supply:demand ratio as severe stress marker | Captures host stress and kernel vulnerability |
| Rainfall instability | Dry spell followed by humidity/rain | Higher instability increases risk | Zambia dry-spell mycobiome shifts | Captures compound stress |
| Toxigenic fungal abundance | ITS/qPCR/metagenomics | Higher abundance increases risk | Aspergillus/Fusarium relative abundance linked to toxins in Zambia | Captures community-level warning |
| Toxin gene load | aflR, aflD, fum1, tri5, pks genes | Higher gene load increases risk | To be extracted across studies | Moves beyond genus-level identification |
| Toxigenic network centrality | Co-occurrence network metrics | Higher centrality increases risk | Proposed review-derived metric | Identifies toxigenic fungi as ecological hubs |
| Antagonist depletion | Atoxigenic Aspergillus, Trichoderma, Bacillus, yeasts | Lower antagonist abundance increases risk | Atoxigenic biocontrol reduced aflatoxin up to 76-100% in Ghana | Quantifies suppression debt |
| Crop injury | Insect damage, drought injury, lodging | Higher injury increases risk | Insect injury promotes fungal entry and spread | Captures entry points and substrate damage |
| Storage amplification | Moisture, drying delay, storage temperature | Higher amplification increases risk | Stutt et al. farm-to-factory modelling | Links pre-harvest signal to food-chain outcome |
| Human exposure link | Urinary/blood/breast milk biomarkers | Higher biomarkers confirm translation | DON detected in 99% of German 24-hour urine samples | Connects crop risk to internal dose |

*Source note:* SMRI is a proposal requiring external validation; variables should be standardized by crop, region, sampling stage, and analytical platform (Battilani et al., 2016; Chauhan et al., 2015; Katati et al., 2023; Agbetiameh et al., 2020).

**Table 8.** Data-generating hypotheses for future One Health studies (Katati et al., 2023; Stutt et al., 2023; Owolabi et al., 2024).

| **Hypothesis** | **Minimum dataset** | **Primary endpoint** | **Expected novel insight** |
| --- | --- | --- | --- |
| SMRI predicts toxin contamination earlier than toxin testing | Climate + crop mycobiome + toxin assay | Time from dysbiosis signal to toxin exceedance | Establishes dysbiosis-before-toxin paradigm |
| Antagonist depletion predicts aflatoxin independent of Aspergillus abundance | Mycobiome + antagonist qPCR + aflatoxin | Aflatoxin concentration | Tests microbiome suppression debt |
| Climate stress changes toxin mixtures, not only toxin quantity | Multi-toxin LC-MS/MS + weather | Mixture burden score | Moves beyond single-toxin risk |
| Pre-harvest SMRI predicts human urinary biomarkers | Field mycobiome + food toxin + urine/blood biomarkers | Biomarker concentration | Connects crop ecology to human exposome |
| Atoxigenic biocontrol lowers human biomarkers | Intervention + crop toxin + human biomonitoring | Change in AFB1-lysine or AFM1 | Converts agricultural biocontrol into One Health intervention |
| Explainable AI identifies biologically meaningful warning signals | Multimodal dataset + SHAP/feature importance | Model interpretability | Builds trust and mechanistic plausibility |
| Infant-food thresholds reveal vulnerability compression | Regulatory limits + exposure modelling | Infant/adult safety compression factor | Shows policy already encodes vulnerability but prediction models often do not |

*Source note: These hypotheses are intended to convert the conceptual framework into testable field, intervention, and biomonitoring designs (Katati et al., 2023; Stutt et al., 2023; Owolabi et al., 2024).*
